# Genetic mapping and molecular mechanism behind color variation in the Asian vine snake

**DOI:** 10.1186/s13059-023-02887-z

**Published:** 2023-03-09

**Authors:** Chen-Yang Tang, Xiaohu Zhang, Xiao Xu, Shijie Sun, Changjun Peng, Meng-Huan Song, Chaochao Yan, Huaqin Sun, Mingfeng Liu, Liang Xie, Shu-Jin Luo, Jia-Tang Li

**Affiliations:** 1 CAS Key Laboratory of Mountain Ecological Restoration and Bioresource Utilization & Ecological Restoration and Biodiversity Conservation Key Laboratory of Sichuan Province, Chengdu Institute of Biology, Chinese Academy of Sciences, Chengdu, 610041 China; 2 Key Laboratory of Birth Defects and Related Diseases of Women and Children, Ministry of Education, West China Second University Hospital, Sichuan University, Chengdu, 610041 China; 3 Sichuan University-The Chinese University of Hong Kong Joint Laboratory for Reproductive Medicine, West China Second University Hospital, Sichuan University, Chengdu, 610041 China; 4 The State Key Laboratory of Protein and Plant Gene Research, School of Life Sciences, Peking University, Beijing, 100871 China; 5 Peking-Tsinghua Center for Life Sciences, Academy for Advanced Interdisciplinary Studies, Peking University, Beijing, 100871 China; 6 University of Chinese Academy of Sciences, Beijing, 100049 China; 7 Center for Excellence in Animal Evolution and Genetics, Chinese Academy of Sciences, Kunming, 650223 China; 8 Southeast Asia Biodiversity Research Institute, Chinese Academy of Sciences, Yezin Nay Pyi Taw, 05282 Myanmar

**Keywords:** Reptiles, Skin color, *Ahaetulla prasina*, Iridophores, GWAS

## Abstract

**Background:**

Reptiles exhibit a wide variety of skin colors, which serve essential roles in survival and reproduction. However, the molecular basis of these conspicuous colors remains unresolved.

**Results:**

We investigate color morph-enriched Asian vine snakes (*Ahaetulla prasina*), to explore the mechanism underpinning color variations. Transmission electron microscopy imaging and metabolomics analysis indicates that chromatophore morphology (mainly iridophores) is the main basis for differences in skin color. Additionally, we assemble a 1.77-Gb high-quality chromosome-anchored genome of the snake. Genome-wide association study and RNA sequencing reveal a conservative amino acid substitution (p.P20S) in *SMARCE1*, which may be involved in the regulation of chromatophore development initiated from neural crest cells. *SMARCE1* knockdown in zebrafish and immunofluorescence verify the interactions among *SMARCE1*, iridophores, and *tfec*, which may determine color variations in the Asian vine snake.

**Conclusions:**

This study reveals the genetic associations of color variation in Asian vine snakes, providing insights and important resources for a deeper understanding of the molecular and genetic mechanisms related to reptilian coloration.

**Supplementary Information:**

The online version contains supplementary material available at 10.1186/s13059-023-02887-z.

## Background

Phenotypic diversity of organisms is the basis of environmental adaptation, as well as survival and reproduction. Color polymorphism is an important component of phenotypic diversity, defined as the presence of two or more morphs with different color phenotypes (color variants) within a population [1]. Color polymorphisms have long fascinated biologists as they can provide a key biological model for the study of sexual selection, speciation, adaptation, and evolution [2].

Squamate reptiles (lizards and snakes) are among the most colorful vertebrate, with color polymorphisms occurring across multiple lineages and species [3]. Reptile coloration and patterning are primarily formed by three types of chromatophores (i.e., melanophores, xanthophores, and iridophores) derived from neural crest cells (NCCs) [4]. The arrangements and interactions of these chromatophores result in different hues and colors [5], which typically indicate specific functions, such as intra-/inter-specific communication, sexual selection, and adaptive survival [6]. NCCs are multipotent embryonic cells that give rise to a vast array of derivatives, including neurons, skeletal components, and chromatophores [7]. Studies on gene regulatory networks (GRNs) governing diversification of these precursors have identified various transcription factors that regulate fate specification of chromatophores from NCCs during developmental process (e.g., *sox10*, *MITF*, and *tfec*) [8, 9].

Previous research on skin color has focused on morphology, biochemistry, and ecology [10, 11], with limited studies exploring the molecular mechanisms underlying proven pigmentation pathways, such as pigment synthesis in chromatophores (melanophores or xanthophores) [12–14]. For example, transposon insertions prevent melanocyte maturation, resulting in a white coat phenotype in buffalo [15]. In addition, changes in the regulatory sequences of core biosynthetic genes control yellow and orange coloration in common wall lizards [16]. Nevertheless, the genetic basis and developmental mechanisms of coloration in vertebrates show considerable diversity [17, 18]. Previous studies have demonstrated that regulatory changes in the development of NCCs into chromatophores can lead to differences in pigmentation [19]. For example, dorsal patterning in the brown anole is influenced by the migration of pigment cells derived from NCCs [20]*.* For the complex process of chromatophore development, however, most studies have applied on zebrafish models in research [21–23]. Thus, our understanding of the role of chromatophore development in wild reptile color variation remains limited.

Here, to explore the molecular basis of skin color polymorphism in reptiles, we investigated Asian vine snakes (*Ahaetulla prasina*), which usually contain green (common), yellow, gray, blue, and brown morphs within the same population [24, 25]. Therefore, we analyzed the variations and potential molecular mechanism of color diversity in Asian vine snakes from the perspective of genetics. We combined transmission electron microscopy (TEM) and metabolomic, genomic, and transcriptomic data to explore the genetic mechanism underlying the green and yellow skin phenotypes of the Asian vine snake. Results revealed that an amino acid substitution (p.P20S) in SWItch/sucrose non-fermentable (SWI/SNF)-related matrix-associated actin-dependent regulator of chromatin subfamily E member 1 (*SMARCE1*) was strongly associated with skin color differences in the yellow morph. Thus, we further explored and verified the influence of *SMARCE1* on zebrafish chromatophore development and investigated the underlying molecular mechanisms in the Caco2 cell line. In conclusion, we identified a potential molecular mechanism responsible for color variation in Asian vine snakes, thus providing an important basis for the study of color polymorphism in reptiles.

## Results

### Morphological and biochemical basis of chromatophores underlying pigmentation differences

**Fig. 1:**
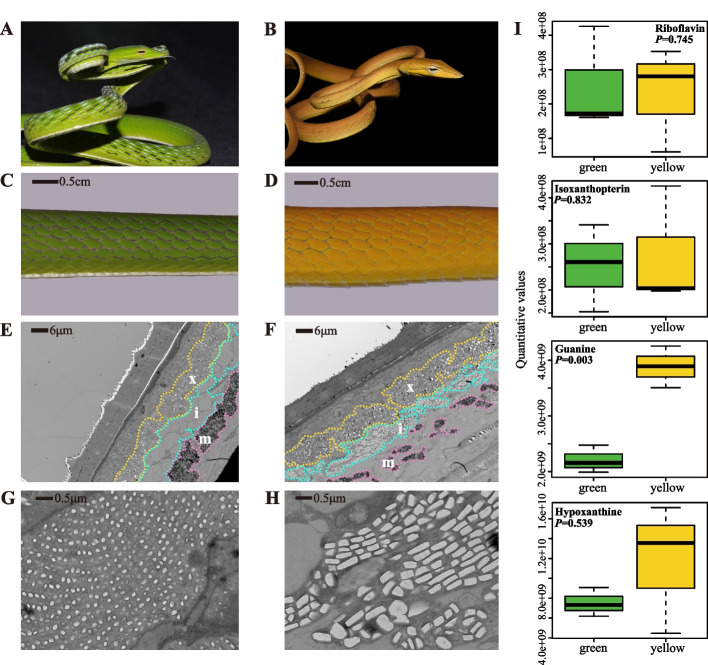
Skin color and cellular differences in two morphs of *Ahaetulla prasina*. **A, B** Green and yellow *Ahaetulla prasina* snakes showing same morphology except for skin color. **C, D** Color details in dorsal skin of two morphs showing distinct differences in hue. **E, F** TEM images of three chromatophore layers with orderly arrangement in dorsal skin, but with differences in iridophore and melanophore cellular morphology. **G, H** Images of different iridophore structures between two morphs. **I** Contents of colored metabolites in skin of both morphs obtained by HPLC-MS/MS. x, xanthophores; i, iridophores; m, melanophores

As green and yellow morphs are the most common among wild Asian vine snakes, we investigated the cellular basis of these pigmentation differences (Fig. 1A–D). TEM imaging showed a consistent arrangement of chromatophores and structural components in both morphs. The chromatophores (i.e., xanthophores, iridophores, and melanophores) demonstrated orderly arrangement in the skin tissue from the epidermis to dermis (Fig. 1E, F, Additional file 1: Fig. S1). However, fewer melanophores were found in yellow individuals, resulting in a thinner melanin layer. Furthermore, the morphs showed marked differences in the iridophore layer, which typically contains light-reflective platelets composed of guanine crystals [26] (Fig. 1G, H, Additional file 1: Fig. S1). Notably, the yellow morphs contained iridophores with disordered and relatively thicker crystal platelets. Based on metabolome analysis, we also explored the composition and content of metabolites in the skin of both morphs. In agreement with the TEM results, we found no significant differences in the content of carotenoids and pterins, but guanine was significantly higher in the yellow morphs, with higher hypoxanthine content (Fig. 1I, Additional file 1: Table S1). These findings indicate that differences in the distribution and density of chromatophores, especially iridophores, may be responsible for the obvious skin color variations in Asian vine snakes.

### High-quality genome assembly of Asian vine snake

**Fig. 2:**
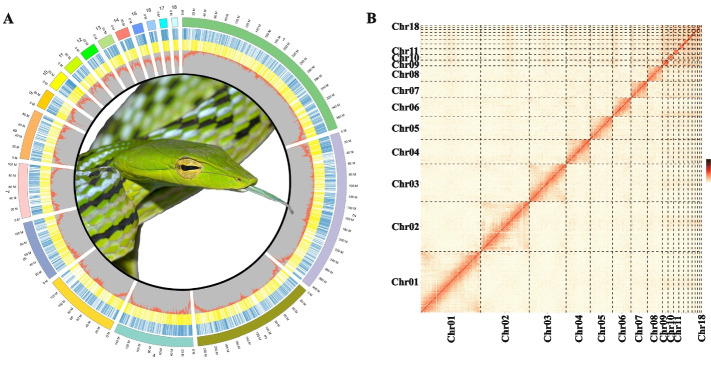
Genomic landscape of *Ahaetulla prasina*. **A** Circos diagram of *Ahaetulla prasina* genome characteristics. Numbers 1 to 18 refer to chromosomes sequenced, with circular rings outside to inside referring to coding genes of plus (blue) and minus (yellow) strand, and GC content per 100-kb window, respectively. **B** Hi-C interactions among 18 chromosomes of *Ahaetulla prasina*. Darker color indicates stronger interactions

We next sequenced and assembled a high-quality reference genome of the Asian vine snake. Long reads (~80 × coverage) produced by Nanopore sequencing were used to assemble the raw genome, while short reads (~60 × coverage) were used to polish the genome assembly. The final chromosome-level reference genome was then assembled using Hi-C data. This yielded 18 scaffolds ranging in size from 11.52 to 377.63 Mb, matching the species karyotype (2n = 36) (Additional file 1: Table S2, Additional file 1: Fig. S2, and Fig. 2A, B). A high-quality chromosome-scale assembly was thus obtained, with a size of 1.77 Gb, contig N50 of 23.87 Mb, and scaffold N50 of 231.95 Mb (Additional file 1: Fig. S3, Additional file 1: Table S3). Genome assembly quality was assessed using benchmarking universal single-copy orthologs (BUSCO) with genome mode and lineage data from vertebrates (Additional file 1: Table S4). Genome completeness (92.8%) was comparable to that of other relatives assembled using various sequencing platforms and assemblers (Additional file 1: Table S5). The genome assembly was annotated using *de novo*-, homology- and transcriptome-based methods, which predicted 18,362 protein-coding genes (Additional file 1: Table S6). This high-quality genome has provided important support for subsequent genomic analysis.

### Mapping of SNP variants for yellow morphs

To obtain genomic polymorphism information on the yellow and green morphs, 60 Asian vine snakes (30 individuals for each color) were re-sequenced, with an average coverage of ~15-fold (Additional file 1: Table S7). The produced reads were aligned to the reference genome to identify single nucleotide polymorphisms (SNPs), genotype, and allele frequency. After quality control, we obtained a total of 12,562,549 SNPs. Based on all SNPs, a maximum-likelihood (ML) tree was constructed, which showed that the color phenotype had no effect on genetic structure (Additional file 1: Fig. S4).

**Fig. 3:**
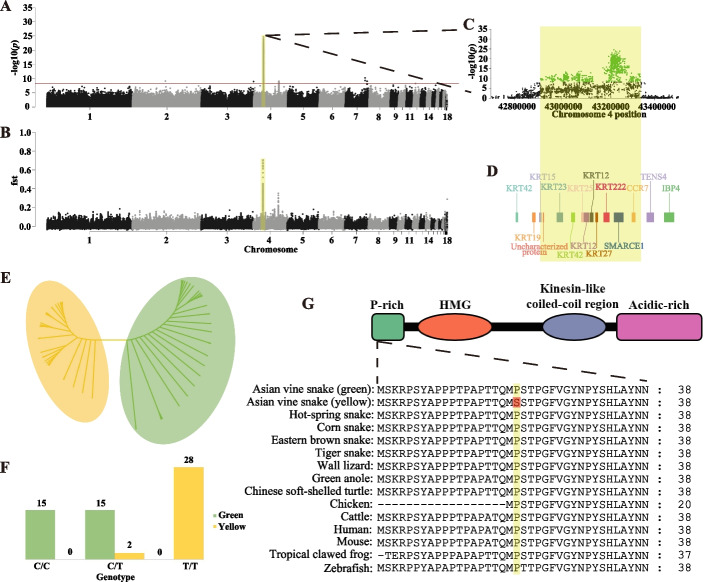
Whole-genome sequencing identification of amino acid substitution P20S in *SMARCE1* on chromosome 4, associated with skin color differences. **A, B** Manhattan plot showing a single region on Chr04, significantly associated with color differences between morphs. Red dash indicates Bonferroni-corrected critical *p*-value (−log_10_(*p*) = 8.4). **C** Association signals of GWAS analysis and genetic differentiation (Fst) in significant signal region. **D** Gene models within significant signal region. **E **Maximum-likelihood tree constructed by SNPs within significant signal region. **F** Correlations between *Ahaetulla prasina* skin color phenotypes and genotypes of *SMARCE1* p.P20S. **G** Schematic and partial alignment of SMARCE1 showing location of P20S in an evolutionarily conserved region within a proline-rich structure across vertebrates

We applied a genome-wide association study (GWAS) using Fisher’s exact test with a Bonferroni-corrected *p* < 0.05 threshold. We identified an interval on chromosome 4 that contained 903 genome-wide significant SNPs (-log10 *p* = 8.40) and showed a strong association with the color phenotype (Fig. 3A), with high confidence (Additional file 1: Fig. S5). Population genomic analysis was also performed to detect signatures of selection underlying the yellow phenotype. Genomic scans for population genetic differentiation (measured by F_ST_) were conducted using a sliding window of 10 kb length based on the Linkage disequilibrium (LD) decay rates (Additional file 1: Fig. S6). The results revealed that the region strongly associated with GWAS-based color also carried a signature of high differentiation between the two morphs (Additional file 1: Table S8, Fig. 3B). The region spanned 426.29 kb (Chr04: 42,912,390–43,338,676) (Additional file 2: Table S9, Fig. 3C) and harbored 11 protein-coding genes, including genes of the *Krt* family, *SMARCE1* and *CCR7* (Additional file 1: Table S10, Fig. 3D). We next constructed a phylogenetic tree for the two snake groups using the ML algorithm based on polymorphic SNPs in the signal region (Fig. 3E). The phylogenetic tree revealed that individuals from the different morphs were clustered separately by color. Based on the 903 genome-wide significant SNPs in the signal region, variant annotation and functional prediction identified three missense variants (Additional file 1: Table S11). However, only one variant (Chr04: 432,332,81, c.58C > T, p.P20S) was predicted to have a deleterious impact on proteins (Additional file 1: Table S12). The p.P20S missense mutation occurred specifically in the yellow Asian vine snakes and was positioned within the 3rd exon of the protein-coding gene *SMARCE1*.

To confirm the association between genetic architecture and colorations, we examined genotypic data of the mutant loci. Genotyping revealed that 28 of the 30 yellow individuals were homozygous for the T/T allele, whereas green individuals were either heterozygous (C/T, *n* = 15) or homozygous (C/C, *n* = 15) (Fig. 3F). The two heterozygous yellow-skinned snakes exhibited locus discordance, possibly due to incomplete penetrance or other interacting genetic factors. Analysis confirmed the consistent effects of mutation and specific skin color variation in the Asian vine snakes. Subsequently, we examined a larger cohort of vertebrates to verify the relationship between mutation and function. Results showed that the loci were located in the proline-rich region of SMARCE1 and were evolutionarily conserved across vertebrates (Fig. 3G), suggesting a possible significant impact on protein function. We performed protein structure prediction of the mutant sequence and compared it to the wild-type. Results indicated that the spatial structure of the SMARCE1 protein changed considerably after mutation (Additional file 1: Fig. S7). These findings strongly suggest that *SMARCE1* p.P20S is a crucial mutation in the color variation between green and yellow morphs of Asian vine snakes.

### Downregulation of tfec in skin of yellow morphs

Given that *SMARCE1* is a likely candidate gene for color variation, we conducted RNA sequencing (RNA-seq) of skin samples from the 30 Asian vine snakes (15 of each color morph) to determine the effects on the expression of genes associated with chromatophores (Additional file 1: Table S13). In total, 209 genes were differentially expressed, including the notable chromatophore development-related gene, transcription factor EC (*tfec*) (log_2_fold-change = 2.98; Additional file 1: Fig. S8). *Tfec* is a master gene of iridophore differentiation and chromatophore development [27]. The significant difference in *tfec* expression further highlighted the crucial role of chromatophore development in color variation between the yellow and green morphs. Based on functional enrichment analysis, the most significantly enriched Gene Ontology (GO) terms in the differentially expressed genes (DEGs) were involved in muscle construction (e.g., actin, myosin, and collagen) and calcium regulation ( Additional file 1: Fig. S9). These results suggest that the two morphs differ in their mediation of iridophore structural organization and crystal array architecture.

### Knockdown of SMARCE1 in zebrafish affects chromatophore development

**Fig. 4:**
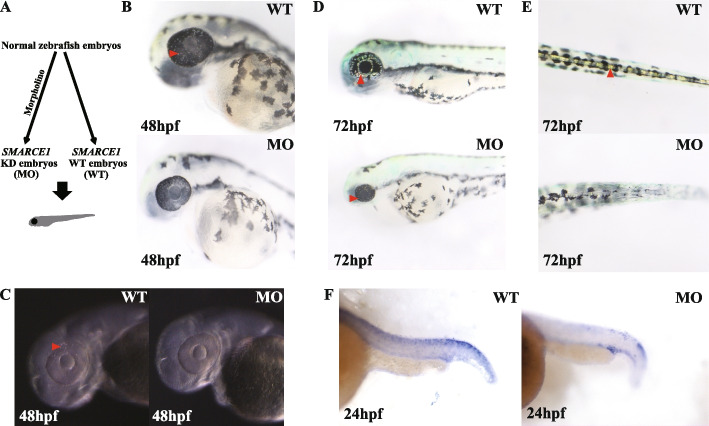
Morpholino-injected embryos show abnormal development of iridophores. **A** Schematic of *SMARCE1* knockdown in zebrafish embryos using morpholinos. **B** Visible iridophores were observed in the eyes of wild-type (WT) embryos but not in morphants (MO) at 48 hpf. **C** Under high-contrast display mode, light-reflecting iridophores were found in WT embryos at 48 hpf. **D** At 72 hpf, morphants contained primary differentiated iridophores, as shown in WT embryos at 48 hpf, while a large number of iridophores were seen in WT eyes. **E** Obvious mature iridophores were observed in dorsal of WT versus morphant embryos at 72 hpf. **F** ISH analysis showed reduced *tfec* expression in tails of morphants at 24 hpf. Arrows indicate visible iridophores. All images were taken under reflected light

To clarify the role of *SMARCE1* in chromatophore development, we generated *SMARCE1*-deficient zebrafish morphants using morpholinos against *SMARCE1* (Fig. 4A). Microscopic observation of zebrafish embryos at different stages showed evident differentiation of iridophores at 48 hours post-fertilization (hpf) in the eyes of the wild-type fish. However, 32 of the 156 injected embryos did not show obvious iridophore differentiation at the same stage (Fig. 4B, C). Based on imaging at 72 hpf, the *SMARCE1* morphants showed reduced iridophores in the eyes, dorsal, and ventral, as well as other phenotypes, such as body curvature, cardiac edema, and smaller eyes (Fig. 4D, E, Additional file 1: Fig. S10). Nevertheless, as the effects of morpholinos are usually short-lived, suppression of mRNA translation only lasted about 3 days, and the iridophores recovered in most *SMARCE1* morphants after 96 hpf. To better judge differences in iridophore development, morpholino-injected embryos were fixed at 24 hpf and *in situ* hybridization (ISH) microscopy was used to assess changes in iridophore master gene (i.e., *tfec*) expression in the dorsal region, with abnormal expression found in the morphants (Fig. 4F). Thus, our results indicated that the *SMARCE1*-deficient morphants exhibited abnormal differentiation of iridophores and lower expression of the chromatophore development-related master gene *tfec*.

### SMARCE1 knockdown blocks recruitment of tfec in Caco2 cells

**Fig. 5:**
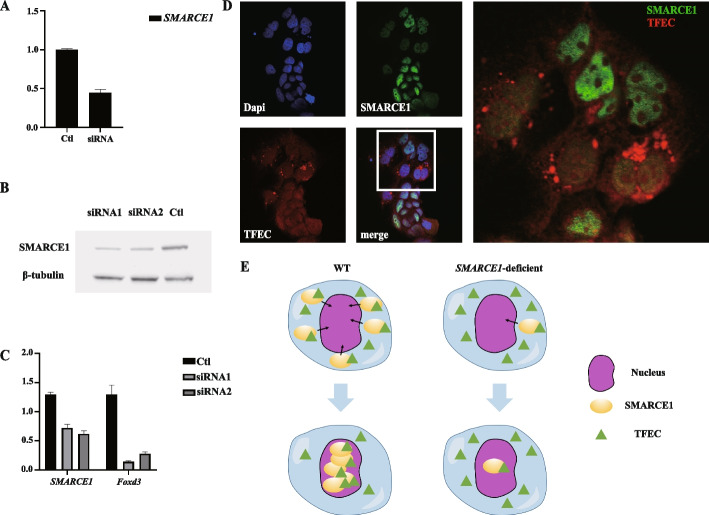
*SMARCE1* deficiency in Caco2 cells blocks recruitment of tfec in nucleus. **A ***SMARCE1* expression in purified Caco2 cell population determined by qRT-PCR. Ctl, control group; siRNA, siRNA-injected group. **B** Immunoblotting analysis of SMARCE1 in Caco2 cells. Ctl, control group; siRNA1 and siRNA2, siRNA-injected groups. **C ***SMARCE1* and *Foxd3* expression in purified Caco2 cell population determined by qRT-PCR. Ctl, control group; siRNA1 and siRNA2, siRNA-injected groups. **D** Immunofluorescence staining of SMARCE1 and tfec showing substantial accumulation of tfec fluorescence signals around the nucleus in *SMARCE1*-deficient Caco2 cells. **E** Schematic of possible mechanism by which defective *SMARCE1* impedes *tfec* nuclear recruitment

To study the interactions between *SMARCE1* and *tfec*, targeted small interfering RNA (siRNA) was applied to knockdown *SMARCE1* in Caco2 cells, which constitutively express the SWI/SNF complex and *tfec*. Quantitative real-time polymerase chain reaction (qRT-PCR) and western blotting showed that the mRNA and protein expression levels were both significantly decreased by siRNA treatment (Fig. 5A, B). Interestingly, *Foxd3*, a key gene related to NCC differentiation [28], was also downregulated after *SMARCE1* knockdown (Fig. 5C). Immunofluorescence co-staining of SMARCE1 and tfec revealed that the expression level of *SMARCE1* was significantly decreased in about 50% of the Caco2 cells treated with *SMARCE1*-siRNA (Fig. 5D). In cells showing low *SMARCE1* expression, the tfec signal was aggregated and localized in the cytoplasm rather than the nucleus. However, in those cells without *SMARCE1* knockdown, *SMARCE1* was significantly expressed in the nucleus and co-localized with high tfec signals (Fig. 5D). These results suggest a potential functional interaction between *SMARCE1* and *tfec*. Furthermore, knockdown of *SMARCE1* may prevent nuclear translocation of *tfec* and block its downstream gene expression (Fig. 5D, E).

## Discussion

Exploring the genetic foundation of animal coloration differences provides an important perspective for studying phenotypic diversity, species differentiation, and adaptive evolution [29]. Although reptiles exhibit considerable skin color variation within species, the underlying molecular and genetic mechanisms remain largely unknown, partly hindered by a lack of molecular data [30]. Thus, high-quality chromosome-anchored genome assemblies should facilitate studies on color variations [15]. By combining whole-genome sequencing, gene expression analysis, metabolomics, and TEM imaging, we investigated the molecular mechanisms of color variation in Asian vine snakes to suggest a possible genetic basis of vivid reptile coloration.

In vertebrates, both early developmental and physiological changes can lead to differences in chromatophores [18, 21, 31, 32]. Mammals and birds use pigments to color keratinous tissues, such as hair, fur, feathers, and beaks [33, 34]. For instance, changes in melanin pigmentation in tiger hair can alter coat color and stripes [35]. The relative abundances of pteridine and carotenoid in xanthophores also differ among the different color morphs of Australian tawny dragon lizards [13]. In the Asian vine snakes, however, the most obvious cellular difference was not in the melanophores or xanthophores but in the iridophores, which are the main component of structural color (Fig. 1E–H). Multiple thin-layer interference occurs in the reflective platelet layers mainly composed of guanine and hypoxanthine crystals in iridophores [17]. Higher purine content results in larger crystal reflective platelets in iridophores that reflect longer wavelength light [36, 37]. Guanine and hypoxanthine as major differential metabolites in the two different skin morphs, may indicate differences in iridophores in the Asian vine snakes (Fig. 1I, Additional file 1: Table S1). By actively tuning the crystal lattice in the dermal iridophores, chameleons can shift their skin color via changes in the wavelength of reflected light [38]. We discovered a similar structural pattern in the Asian vine snakes, where larger and disordered crystal platelets observed in the iridophores predicted longer wavelengths of yellow reflected light, resembling the crystal platelets in the yellowish inter-stripes of zebrafish [39]. Moreover, the slight increase in melanocytes in the green snake skins may indicate enhancement of differentiation in iridophores with ordered crystals [39]. Cellular morphology of the yellow and green snakes showed that the iridophores play vital roles in the color differences between the two morphs.

The simultaneous morphological differences in iridophores and melanophores between the two different colored Asian vine snake morphs suggest possible effects on chromatophore development [40]. NCCs participate in the initial biological processes of chromatophores [41]. For example, during migration and maturation of chromatophores from NCCs, lysosomal changes in lavender corn snake morphs can influence the morphology of the three types of chromatophores [42]. Recently, several studies have suggested that the large adenosine triphosphate (ATP)-dependent chromatin remodeling complex SWI/SNF plays a key role in neural crest induction, differentiation, and chromatophore development [43, 44]. The SWI/SNF complex can alter chromatin structure and regulate transcription [45]. *SMARCE1* plays a vital role in the organization, function, and recruitment of the SWI/SNF complex [46–48]. Moreover, *SMARCE1* can interact with the melanocyte-inducing transcription factor (*MITF*) to promote the expression of distinct *MITF* target genes [49], which is vital for melanocyte differentiation [50]. These studies highlight the important link between *SMARCE1* and chromatophore development, especially the interactions with the master regulator driving chromatophore specification.

Based on our GWAS analyses of color morphs, we found a conservative missense mutation, i.e., p.P20S, within the coding sequence of *SMARCE1* in the yellow morphs, which is a crucial component of the evolutionarily conserved SWI/SNF complex (Fig. 3A–D, G). Most yellow snakes were homozygotes based on the SNP loci, suggesting a key effect of mutation on skin color differences (Fig. 3F). The existence of the SMARCE1 subunit likely functions to maintain proper subunit composition of the SWI/SNF complex [51] and the N-terminal proline-rich region of SMARCE1, where the mutation is located, is important for complex assembly [45]. Here, protein variation analysis and structural prediction likewise verified the significance of the p.P20S to SMARCE1 (Additional file 1: Table S12, Additional file 1: Fig. S7). Furthermore, the *SMARCE1* knockout zebrafish suggested the involvement of *SMARCE1* in NCC differentiation, as the microphthalmia phenotype in *SMARCE1*-deficient embryos might indicate disruption of the NCC deriving process [52]. Similar abnormal phenotypes occurred in our *SMARCE1* morphant embryos, with abnormal body curvature, smaller eyes, and cardiac edema indicating strong interference with NCC development (Additional file 1: Fig. S10). In addition, abnormal iridophores appeared in the eyes and body of morphants during early embryonic development (Fig. 4B–E). These results suggest that *SMARCE1* is involved in NCC differentiation, especially the development of chromatophores. Thus, these findings expand our understanding of the important role of NCC differentiation in pigmentation differences.

In zebrafish, the differentiation of NCCs into chromatophores occurs at the very early stage of embryonic development [53], with the master *tfec* gene playing a vital role in progressive fate restriction of chromatophores [9], especially the iridophore lineage [27]. In the current study, we found that *tfec* expression was markedly lower in morphants than in wild-type variants at 24 hpf (Fig. 4F), indicating that chromatophore differentiation was affected by *SMARCE1* knockdown, showing consistency with skin transcriptome analysis in the different colored snakes (Additional file 1: Fig. S8). Notably, functional enrichment analysis also identified changes in the chromatophores of the Asian vine snakes. As actin and myosin filaments play roles in multiple chromatophore cell processes, such as motility, morphological changes, and structural organization of iridophores [54, 55], the DEG-enriched functions between the two morphs provide further clues regarding different iridophore morphogenesis (Additional file 1: Fig. S9).

Finally, we identified strong interactions between *SMARCE1* and *tfec*. *Tfec* belongs to the MiT family of transcription factor regulators [56] and is the master regulator driving iridophore specification from multipotent progenitors [9]. As a paralogous gene of *MITF*, the master regulator of melanocyte fate, *tfec* shows similar expression patterns to *MITF* in eyes of zebrafish [19, 57]. Here, in the *SMARCE1*-deficient Caco2 cells, tfec recruitment to the nucleus was hindered, possibly due to the attenuation of tfec and SWI/SNF complex binding, resulting in the poor biological function of *tfec* (Fig. 5D, E). Of note, *Foxd3*, an essential upstream regulator of neural crest determination, was downregulated in the *SMARCE1*-deficient Caco2 cells (Fig. 5C). These results support our hypothesis that the *SMARCE1* mutation generates abnormal *tfec* function in NCC differentiation and ultimately affects chromatophore development, especially that of iridophores.

In addition, we speculated that Asian vine snakes with other colors similarly follow this pattern of differences in iridophore morphology, and subtle differences in xanthophore and melanophore morphology may be the potential mechanisms for the formation of the other skin colors rather than green or yellow. Further researches are needed to verify the possibilities.

## Conclusions

In summary, we assembled a high-quality reference genome and used multi-omics to reveal the genetic underpinning for color variations in the Asian vine snake. Our research indicated that *SMARCE1* is important for chromatophore normal development especially iridophores derived from NCCs, which is likely to be the underlying mechanism of the color variations in Asian vine snakes. The findings will enable future genetic and evolutionary research on skin color polymorphism in reptiles. Indeed, different color morphs of Asian vine snakes coexist in the wild, but the ecological fitness of the skin color variations remain unclear. Additional observation and analyses are warranted to identify the adaptive significance of color variation.

## Methods

### Ethics statement

The authors followed all appropriate ethical and legal guidelines and regulations. The Chengdu Institute of Biology, Chinese Academy of Sciences (CIB, CAS), facilitated the collection and exportation permits necessary for this and related studies. The study was approved by the Animal Ethics Committee of Chengdu Institute of Biology, Chinese Academy of Sciences (CIBDWLL20202632, 13 April 2020). Research procedures were carried out in accordance with all national and institutional regulations.

### Phenotypic characterization

All specimens used for sequencing and experimentation were collected from Yunnan Province, China. All vouchers are stored in the Herpetological Museum of the Chengdu Institute of Biology, Chinese Academy of Sciences.

For the TEM experiments, two fresh skin samples (1cm×1cm) per color morph from the Asian vine snake were collected. The samples were then cut into small pieces (1 mm^3^) in fixative. The tissue blocks were transferred to an Eppendorf tube with fresh TEM fixative for further fixation, then washed using 0.1 M PB (pH 7.4) three times (15 min each). The samples were dehydrated in an increasing ethanol series at room temperature, followed by two changes of acetone and transfer to resin for embedding. The resin blocks were cut to 60–80-nm slices on an ultra-microtome (Leica, UC7), and the ultra-thin sections were put onto the 150-mesh cuprum grids. The cuprum grids were then stained with 2% uranium acetate-saturated alcohol solution and 2.6% lead citrate, respectively. Finally, the cuprum grids were observed under a TEM (Hitachi, HT7800/HT7700) and imaged.

### Metabolite analyses

We prepared three fresh skin samples per color morph for metabolomics analysis. Metabolite content in the skin of both morphs was determined using high-performance liquid chromatography-tandem mass spectrometry (HPLC-MS/MS). Tissues (100 mg) were individually ground with liquid nitrogen and the homogenate was resuspended and well-vortexed in prechilled 80% methanol and 0.1% formic acid (FA). After centrifugation at 15,000 rpm and 4°C for 5 min, the resulting supernatant was diluted to a final concentration containing 53% methanol using LC-MS-grade water. The samples were subsequently transferred to a fresh Eppendorf tube and centrifuged at 15,000*g* and 4°C for 10 min. Finally, the supernatant was added to the Vanquish UHPLC system (Thermo Fisher Scientific, USA) coupled with an Orbitrap Q Exactive series mass spectrometer (Thermo Fisher Scientific, USA) for analyses. Samples were injected onto a Hypersil Gold column (100×2.1 mm, 1.9μm) using a 16min linear gradient at a flow rate of 0.2mL/min. The eluents used for positive polarity mode were eluent A (0.1% FA in water) and eluent B (methanol). The eluents for the negative polarity mode were eluent A (5 mM ammonium acetate, pH=9.0) and eluent B (Methanol). The solvent gradient was set as follows: 2% B, 1.5 min; 2–100% B, 12.0 min; 100% B, 14.0 min; 100–2% B, 14.1 min; 2% B, 17 min. The Q Exactive series mass spectrometer was operated in positive/negative polarity mode with spray voltage of 3.2 kV, capillary temperature of 320°C, sheath gas flow rate of 35 arb and aux gas flowrate of 10 arb. The raw data generated by UHPLC-MS/MS were processed using Compound Discoverer 3.1 (CD3.1, Thermo Fisher Scientific, USA) to perform peak alignment, peak picking, and quantification of each metabolite. After that, peak intensities were normalized to total spectral intensity. The normalized data were used to predict the molecular formula based on additive ions, molecular ion peaks, and fragment ions. Accurate qualitative and relative quantitative results were obtained by matching the peaks with the mzCloud (https://www.mzcloud.org/), mzVaultand MassList databases.

All metabolites were annotated using the Kyoto Encyclopedia of Genes and Genomes (KEGG) database (http://www.genome.jp/kegg/), Human Metabolome Database (HMDB) database (http://www.hmdb.ca/), and Lipidmaps database (http://www.lipidmaps.org/). We applied univariate analysis (*t*-test) to calculate statistical significance (*p*-value). Metabolites with *p* < 0.05 and fold-change ≥ 1.5 or ≤ 0.5 in analysis were considered significantly differential metabolites.

### Genome sequencing

To generate a reference genome sequence of the Asian vine snake, muscle tissue from a male green snake (ID: CIB119038) from Xishuangbanna, Yunnan Province, China, was collected. High molecular weight genomic DNA was prepared using the CTAB method, followed by purification using a QIAGEN® Genomic kit (QIAGEN, Valencia, CA, USA) for sequencing according to the standard procedures provided by the manufacturer.

For genome sequencing, DNA was extracted using the SDS method. DNA degradation and extracted DNA contamination were monitored using 1% agarose gels. DNA purity was then detected using a NanoDrop™ One UV-Vis Spectrophotometer (Thermo Fisher Scientific, USA), with OD 260/280 ranging from 1.8 to 2.0 and OD 260/230 ranging from 2.0 to 2.2. Lastly, the DNA concentration was further measured using a Qubit® 4.0 Fluorometer (Invitrogen, USA). In total, 3–4 μg of DNA per sample was used as input material for the ONT library preparations. After the sample was qualified, size selection of long DNA fragments was performed using the PippinHT system (Sage Science, USA). The DNA fragments ends were then repaired, and A-ligation reaction was conducted using a NEBNext Ultra II End Repair/dA-tailing Kit (Cat# E7546). The adapter in SQK-LSK109 (Oxford Nanopore Technologies, UK) was used for further ligation reactions and the DNA library was measured using a Qubit® 4.0 Fluorometer (Invitrogen, USA). A DNA library (700 ng) was constructed and long-read sequencing was performed on a Nanopore PromethION sequencer (Oxford Nanopore Technologies, UK).

For short-read sequencing, a paired-end library was conducted with an insert size of 300 bp and 100 bp paired-end reads, then sequenced using the MGISEQ-2000 platform following the manufacturer’s standard protocols.

For Hi-C sequencing, muscle cells from the Asian vine snake were fixed with formaldehyde, followed by restriction enzyme digestion. Nuclei were extracted by lysing the cross-linked tissue. The cohesive ends were filled in by adding biotinylated nucleotides, and the free blunt ends were ligated. The cross-linking was reversed, and DNA was purified to remove proteins. The purified DNA was then sheared to a length of ∼400 bp and point ligation junctions were pulled down. The Hi-C libraries were sequenced using the Illumina HiSeq platform with PE150 short reads.

### Genome assembly

NextDenovo v1.0 (https://github.com/Nextomics/NextDenovo) was used with parameters “read_cuoff = 2k; seed_cutoff = 20k; blocksize = 2g” to align the long reads sequenced by Nanopore PromethION platform against themselves for self-correction and comparison of overlapping regions to generate consensus sequences to obtain primary assembled genome sequence information. The genome was then further assembled with corrected reads using wtdbg2 [58] with parameters “-k 0 -p 19 -S 2 --rescue-low-cov-edges；wtdbg-cns -c 0 -k 11”. After quality control, the short-read data were aligned to the assembled genome using BWA with default parameters. Contigs were polished using NextPolish v1.01 [59] with three rounds of alignment for long reads, followed by four rounds for short reads. The Hi-C data filtered with fastp v0.20.0 [60] were then used to correct and assemble the contigs to chromosome-level scaffolds using bowtie2 v2.3.2 [61] based on the interaction signals by LACHESIS (http://shendurelab.github.io/LACHESIS/). The completeness of the genome was evaluated against vertebrate lineages with Benchmarking Universal Single-Copy Orthologs (BUSCO v3.1.0).

### Genome annotation

The snake used for genome sequencing was also sampled for genome annotation. RNA was extracted from the heart, lung, and kidney using TRIzol reagent (Invitrogen) for all samples. PolyA mRNA was isolated using oligonucleotide (dT) magnetic beads and disrupted into short segments, and cDNA was synthesized using random hexamer primers and reverse transcriptase. After purification using the RNeasy Mini Kit (QIAGEN), the isolated cDNA was sequenced separately on the Illumina HiSeq 2000 platform. Adapters and low-quality reads were removed to obtain clean reads, which were prepared for coding gene prediction.

We first annotated the tandem repeats using GMATA [62] and Tandem Repeats Finder (TRF) [63] where GMATA identifies simple repeat sequences and TRF recognizes all tandem repeat elements in the whole genome. Transposable elements (TE) in the genome were then identified using both ab initio and homology-based approaches. Briefly, an ab initio repeat library for the Asian vine snake was firstly predicted using MITE-hunter [64] and RepeatModeler [65] with default parameters. The obtainted library was then aligned to the TEclass Repbase (http://www.girinst.org/repbase) to classify the type of each repeat family. For further identification of repeats throughout the genome, RepeatMasker [65] was applied to search for known and novel TEs by mapping sequences against the de novo repeats library and Repbase TE library. Overlapping TEs belonging to the same class were collated and combined.

For gene prediction, three independent approaches, including ab initio prediction, homology search, and reference guided transcriptome assembly, were applied in the repeat-masked genome. In detail, for homology prediction, GeMoMa [66] was used to align the homologous peptides from related species (*Ophiophagus hannah*, *Python bivittatus*, *Thamnophis sirtalis*, *Protobothrops mucrosquamatus*, *Pseudonaja textilis*, and *Notechis scutatus*, Additional file 1: Table S6) to the assembly and gene structure information was obtained. For RNA-seq-based gene prediction, clean reads were aligned to the reference genome using STAR [67]. The transcripts were then assembled using StringTie and open reading frames (ORFs) were predicted using PASA [68]. For de novo prediction, RNA-seq reads were de novo assembled using StringTie and analyzed with PASA to produce a training set. Augustus [69] with default parameters was then used for ab initio gene prediction with the training set. EVidenceModeler [68] (EVM) was used to produce an integrated set of genes with TEs removed using TransposonPSI [70], with the miscoded genes further filtered. Untranslated regions (UTRs) and alternative splicing regions were determined using PASA based on assemblies. We retained the longest transcripts for each gene, and regions outside the ORFs were designated UTRs.

We functionally annotated the coding genes of the newly assembled genome using BLASTP search (*E*-value ≤1*e*−5) against the NR (nonredundant protein sequences in NCBI), SwissProt, and KEGG databases. The SwissProt BLASTP results were processed using our in-house Perl script to retrieve associated GO terms from the idmapping database (accessed on 9 July 2020). All database search results were concatenated.

Two strategies were used to obtain the non-coding RNA (ncRNA): i.e., database searching and model prediction. Transfer RNAs (tRNAs) were predicted using tRNAscan-SE [71] with eukaryote parameters. MicroRNAs, ribosomal RNAs (rRNAs), small nuclear RNAs, and small nucleolar RNAs were detected using the Infernal [72] cmscan program to search against the Rfam database. The rRNAs and their subunits were predicted using RNAmmer [73].

### Whole-genome resequencing

Tail-tip tissue samples of 30 yellow and 30 green morphs were collected, and high-quality DNA was extracted using a QIAGEN® Genomic kit. After monitoring degradation and contamination using 1% agarose gels, DNA purity was checked using a NanoPhotometer® spectrophotometer (IMPLEN, CA, USA). DNA concentration was measured using a Qubit® DNA Assay Kit in Qubit®2.0 Fluorometer (Life Technologies, CA, USA). In total, 700 ng of DNA per sample was used as input material for DNA sample preparation. Sequencing libraries were generated using a NEB Next® Ultra DNA Library Prep Kit for Illumina® (NEB, USA) following the manufacturer’s recommendations and index codes were added to attribute sequences to each sample. The PCR products were purified (AMPure XP system), and library quality was assessed on the Agilent Bioanalyzer 2100 system. The clustering of the index-coded samples was performed using cBot Cluster Generation System with a NovaSeq 6000 PE Cluster Kit (Illumina) according to the manufacturer’s instructions. After cluster generation, the prepared library was sequenced on the Illumina NovaSeq 6000 platform and 150bp paired-end reads were generated.

### Variant detection

Quality control was produced of initial data. Contaminated joints and low-quality reads with N content higher than 5% were removed. Using the assembled Asian vine snake reference genome, the BWA-MEM algorithm [74] was used to align clean reads with the genomic data and the output SAM files were processed using bcftools [75] mpileup with parameters “-q 20 -Q 20 -C 50” and bcftools call with parameters “-f GQ,GP” for SNP identification.

### GWAS and population genomic analysis

We used plink v1.90 [76] for GWAS analysis. The SNP sites with minimum allele frequency (MAF) < 0.1, deletion rate of all individuals > 0.1, and Hardy Weinberg *p* < 10^−5^ were filtered, with green morphs as the control group and yellow morphs as the experimental group. GWAS analysis was performed using Fisher’s exact test with parameters “- assoc fisher”. Genetic differentiation (Fst) between the two morphs was calculated using a sliding window approach (window size 10 kb with step size 5 kb) using VCFtools v0.1.17 [77]. The analysis results were visualized using the R package qqman v0.1.4 [78].

### Variant annotation

SnpEFF [79] v4.3t was applied to annotate each variant using the annotation file in GTF format prepared for the Asian vine snake reference genome. Variant effect prediction was performed using PROVEAN (Protein Variation Effect Analyzer) [80].

### Structural modeling of mutant SMARCE1 protein

Three-dimensional structural models of wild-type and mutant SMARCE1 were predicted based on the whole sequence using AlphaFold2 v2.0.0 in casp14 mode [81]. Pairwise TM-scores of the predicted tertiary structures were calculated with TM-align [82, 83], with the mutant protein of SMARCE1 compared against that of the wild-type using Student’s *t* test to determine whether variation in conformation existed between the models. Structures with a TM-score > 0.5 assume generally the same fold.

### Transcriptome sequencing and analysis

Skin samples were collected from 15 yellow and 15 green morphs. Total RNA was extracted using a QIAGEN® RNA Mini Kit. After monitoring degradation and contamination using 1% agarose gels, RNA purity was checked using a NanoPhotometer® spectrophotometer (IMPLEN, CA, USA). RNA concentration was measured using a Qubit® RNA Assay Kit and Qubit® 2.0 Fluorometer (Life Technologies, CA, USA). RNA integrity was assessed using the RNA Nano 6000 Assay Kit with the Agilent Bioanalyzer 2100 system (Agilent Technologies, CA, USA). In total, 1.5μg of RNA per sample was used as input material for RNA sample preparations. Sequencing libraries were generated using a NEBNext® UltraTM RNA Library Prep Kit for Illumina® (NEB, USA) following the manufacturer’s recommendations, and index codes were added to attribute sequences to each sample. The clustering of the index-coded samples was performed on a cBot Cluster Generation System using a NovaSeq 6000 PE Cluster Kit (Illumina) according to the manufacturer’s instructions. After cluster generation, the library was sequenced on the Illumina NovaSeq 6000 platform and 150bp paired-end reads were generated. After quality control of reads data, a reference genome index was built and high-quality RNA-seq reads were aligned to the reference genome using HISAT2 v2.1.0 [84]. StringTie v2.0.4 [85] was used for transcript assembly and expression level analysis of coding gene. Differential expression analysis was conducted using the R package DESeq2 v1.20.0 [86] based on the read count numbers. The significance of gene expression differences was determined using the Wald test, with |log_2_fold-change|≥1 and *p*-adj<0.05 considered noteworthy. GO and pathway enrichment analyses were then performed and coding genes related to skin coloration and their biological functions were identified using the R package clusterProfiler v3.10.1 [87].

### SMARCE1 knockdown in zebrafish embryos using morpholino

Embryos were obtained by natural mating and cultured in embryo medium. The staging of embryos was carried out according to Kimmel et al [88]. *SMARCE1*-specific morpholino oligonucleotides (5′ - CGCTTTGACATCTTGATTGTAGGGT - 3′) were obtained from Gene Tools (Philomath, OR, USA). Morpholinos were injected at a concentration of 0.5 ng. The injection dose was an estimated amount received by a single embryo. At different post-fertilization stages, the wild-type (WT) and morpholino (MO) embryo groups were imaged using a microscope (Nikon SMZ18, Japan), including chromatophores in the eyes, dorsal, and ventral. We compared the chromatophore density of these three parts between two groups at the same stage and in the same field of view based on microscopy imaging.

### In situ hybridization

Whole-mount in situ hybridization was carried out as described previously in Thisse et al. [89] and Sun et al [90]. The probe DNA template was amplified from the embryonic genome or cDNA using primers (GAGCTGGAGATCCAGGCTCAT, GAAATTAATACGACTCACTATAgggagacccGAAACGGGAGGTCATTCTGAGAGT). Antisense probe RNAs for in situ hybridization were synthesized using a DIG RNA Labeling Kit (SP6/T7) (Roche) and purified using MEGAclear (Ambion).

### Cell culture

Caco2 cells were cultured at 37 °C under 5 % CO_2_ in Dulbecco’s modified Eagle’s medium/Nutrient Mixture F-12 (DMEM/F12) (Gibco, USA) and 10% fetal bovine serum (FBS). The Caco2 cells were then subcultured in a 6-well plate to 80% confluency. Both riboFECT CP reagent (RIBOBIO, Guangzhou, China) and 100 nM siRNA (CCGCGTACCTTGCTTACATAA, CCCATACCAGAAGATGAGAAA) were added to the culture medium and incubated for 48 h before harvesting the cells.

### RNA extraction and cDNA synthesis

Total RNA was isolated from the transfected cells with TRIzol reagent (Invitrogen, Carlsbad, CA, USA) according to the manufacturer’s instructions. Reverse transcription was performed using a Revert Aid First Strand cDNA Synthesis Kit (Thermo, Waltham, MA, USA) according to the manufacturer’s instructions. Total RNA, Oligo (dT)18 primer, Random Hexamer primer, water (nuclease-free), 5×Reaction Buffer, RiboLock RNase Inhibitor, 10 mM dNTP mix, and RevertAid M-MuLV RT were used in first-strand cDNA synthesis. The PCR program was as follows: incubation for 5 min at 25°C, followed by 60 min at 42°C, with the reaction terminated by heating at 70°C for 5 min.

### Quantitative real-time PCR (qRT-PCR)

Here, qRT-PCR was performed to compare mRNA levels of *SMARCE1* and *Foxd3* in transfected and control Caco2 cells, with β-actin used as the reference gene, using TB Green™Premix Ex Taq™II (TaKaRa, Shiga, Japan). The reactions contained 3μL of cDNA, 12.5μL of TB Green Premix Ex Taq II, 1μL of for each primer (from 10 μmol/L stock), with ultra-pure water to a volume of 25μL. PCR was performed on the CFX96 Real-Time PCR Detection System (Bio-Rad, Hercules, CA, USA). The PCR conditions for *SMARCE1*, *Foxd3*, and β-actin were one cycle of 30 s at 95°C followed by 40 cycles of 5 s at 95°C and 1 min at 60°C. Melting curve analysis was conducted from 65 to 95°C, with plate readings taken at each 0.5°C increment. Each analyzed sample was technically duplicated. The sequences of primers used are listed in Additional file 1: Table S14.

### Western blotting

The transfected control Caco2 cells were lysed in RIPA buffer (150 mM NaCl; 50 mM Tris-Cl, pH 8; 1% NP-40; 0.5% deoxycholate; 0.1% SDS) (Beyotime, Shanghai, China) with Protease Inhibitor Cocktail (100×) (Thermo Scientific, MA, USA). The resulting samples were separated using 10% SDS-PAGE (Invitrogen, Carlsbad, CA, USA) and transferred to polyvinylidene fluoride (PVDF) membranes. After blocking for 1 h in 5% non-fat milk in TBST at 25°C, the membranes were incubated overnight at 4 °C with specific primary antibodies, including SMARCE1 (Abcam, Cambridge, UK, 1:1000), tfec (Santa Cruz, USA,1:500), and β-tubulin (Sigma, USA, 1:10,000). Primary antibody binding was visualized using horseradish peroxidase-conjugated goat anti-rabbit or anti-mouse IgG (Sigma, USA, 1:10,000) for 1 h at 25°C. Signal intensities were measured using a Chemiluminescent HRP substrate (Yamei, Shanghai, China) and imaged using ImageJ v1.8.0 (NIH, Bethesda, MD, USA).

### Immunofluorescence staining

Lab-Tek Chamber Slide w/Cover Glass Slide Sterile (Thermo, Waltham, MA, USA) was used to culture and transfect Caco2 cells, with 100 μL of medium per well. Primary antibodies of SMARCE1 (Abcam, Cambridge, UK, 1:100) and tfec (Santa Cruz, USA, 1:50) were used for double immunofluorescence. The cells were fixed with 4% formalin and incubated with specific primary antibodies overnight at 4°C. Primary antibody binding was visualized using fluorescein-labeled donkey anti-rabbit or anti-mouse IgG (Invitrogen, Carlsbad, CA, USA, 1:500) for 1 h at 25°C. DAPI (Solarbio, Beijing, China) was used for nuclear staining. Images were captured using a confocal laser scanning microscope (Olympus FV1000, Japan).

## Supplementary Information

**Additional file 1: Fig. S1.** Supplementary ultrastructure of skin samples of green and yellow morphs. **Fig. S2.** Synteny tracker of chromosomes among genomes of *Naja naja*, *Thermophis baileyi, Ahaetulla prasina*, *Thamnophis elegans*, and *Zootoca vivipara*. **Fig. S3.** Genome complexity assessment. **Fig S4.** Maximum-likelihood tree based on whole-genome SNPs. **Fig. S5.** QQ-plot of all SNPs based on *p*-value in GWAS analysis. **Fig. S6.** Linkage Disequilibrium decay rates of *A.prasina*. **Fig. S7.** The predicted spatial structure comparison between wild-type and mutant proteins of SMARCE1. **Fig. S8.** Volcano diagram of gene expression level. **Fig. S9.** Dotplot of statistically significant GO terms enriched by DEGs. **Fig. S10.** Morpholino injected and wild-type embryos at 72 hpf. **Fig. S11.** The uncropped western blot membranes that showed in Fig. 5B. **Table S1.** Information on the content of main chromatophore-related metabolites. **Table S2.** The chromosome length of *Ahaetulla prasina.***Table S3.** Statistics for *Ahaetulla prasina* genome assemblies*.***Table S4.** Statistics of the completeness genomes using BUSCO. **Table S5.** Quality metrics for *A. prasina* genome compared to other published snake genomes. **Table S6.** Statistics of the annotated genes. **Table S7.** Overview of whole genome sequencing data. **Table S8.** The top 30 genomic windows of population differentiation (Fst). **Table S10.** Coding genes within the region of GWAS signals. **Table S11.** Annotation of 3 missense variants using snpEFF software. **Table S12.** Summary of protein variation effect prediction. **Table S13.** Summary of RNA-seq data from 30 skin samples. **Table S14.** Primers used in qRT-PCR.

**Additional file 2: Table S9.** Significant SNPs in the chromosomal region of GWAS signals.

**Additional file 3.** Review history

## Data Availability

All data supporting the findings in this study are available within this article and its additional files. The reference genome raw sequencing data, whole-genome sequencing data, and RNA-seq data have been deposited at NCBI Sequence Read Archive under accession number PRJNA926696, PRJNA928843, and PRJNA929071, respectively [91–93]. The genome assembly has been deposited in NCBI GenBank under accession number JAQQSC000000000 as well as in Genome Warehouse, National Genomics Data Center (NGDC) under accession number PRJCA014549 [94, 95]. The metabolomic data in this paper have been deposited in the MetaboLights of EMBL-EBI (https://www.ebi.ac.uk/metabolights/) under accession number MTBLS6927 [96]. Perl scripts used for data analyses conducted as part of this study are available at Github: https://github.com/bioinformaticspcj/coding_gene_function_annotation [97], and Zenodo: 10.5281/zenodo.7549964 [98].
