# Supplementary material for: Genetic mapping and molecular mechanism behind color variation in the Asian vine snake
Source: Genome Biol. 2023 Mar 9;24:46. doi: 10.1186/s13059-023-02887-z (PMC9999515; doi:10.1186/s13059-023-02887-z)
Supplement: Supplementary file 1 — Additional file 1: Fig. S1. Supplementary ultrastructure of skin samples of green and yellow morphs. Fig. S2. Synteny tracker of chromosomes among genomes of Naja naja, Thermophis baileyi, Ahaetulla prasina, Thamnophis elegans, and Zootoca vivipara. Fig. S3. Genome complexity assessment. Fig S4. Maximum-likelihood tree based on whole-genome SNPs. Fig. S5. QQ-plot of all SNPs based on p-value in GWAS analysis. Fig. S6. Linkage Disequilibrium decay rates of A.prasina. Fig. S7. The predicted spatial structure comparison between wild-type and mutant proteins of SMARCE1. Fig. S8. Volcano diagram of gene expression level. Fig. S9. Dotplot of statistically significant GO terms enriched by DEGs. Fig. S10. Morpholino injected and wild-type embryos at 72 hpf. Fig. S11. The uncropped western blot membranes that showed in Fig. 5B. Table S1. Information on the content of main chromatophore-related metabolites. Table S2. The chromosome length of Ahaetulla prasina.Table S3. Statistics for Ahaetulla prasina genome assemblies.Table S4. Statistics of the completeness genomes using BUSCO. Table S5. Quality metrics for A. prasina genome compared to other published snake genomes. Table S6. Statistics of the annotated genes. Table S7. Overview of whole genome sequencing data. Table S8. The top 30 genomic windows of population differentiation (Fst). Table S10. Coding genes within the region of GWAS signals. Table S11. Annotation of 3 missense variants using snpEFF software. Table S12. Summary of protein variation effect prediction. Table S13. Summary of RNA-seq data from 30 skin samples. Table S14. Primers used in qRT-PCR. [file 13059_2023_2887_MOESM1_ESM.docx]

Supplementary Materials for

**Genetic mapping and molecular mechanism behind color variation in the Asian vine snake**

Chen-Yang Tang, Xiaohu Zhang, Xiao Xu, Shijie Sun, Changjun Peng, Meng-Huan Song, Chaochao Yan, Huaqin Sun, Mingfeng Liu, Liang Xie, Shu-Jin Luo and Jia-Tang Li^*^

* Correspondence: lijt@cib.ac.cn (Jia-Tang Li)

**This PDF file includes:**

Supplementary Figs. S1 to S11

Tables S1 to S8, S10 to S14

**Supplementary Figures**

**
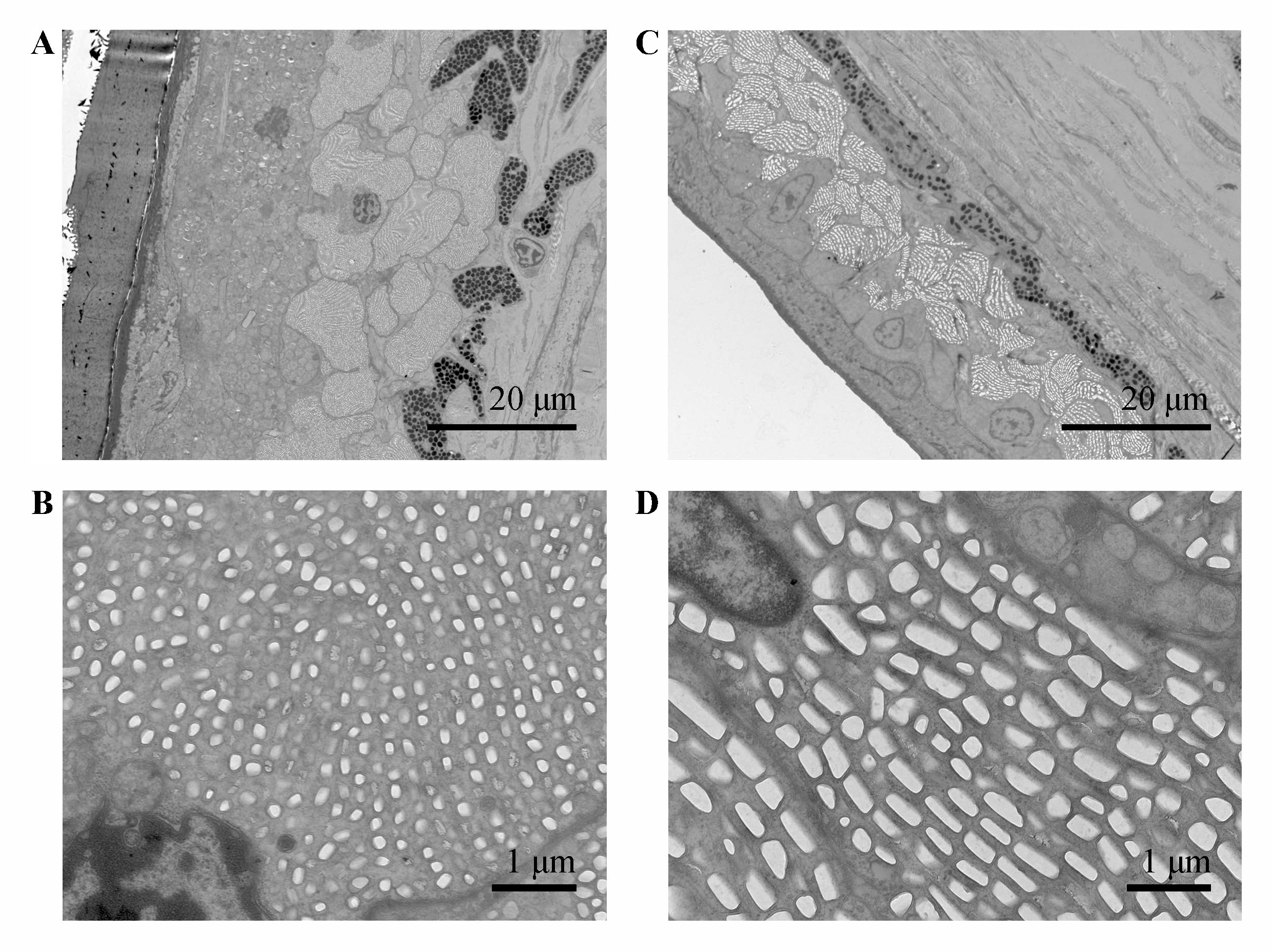
**

**Fig. S1. Supplementary ultrastructure of skin samples of green and yellow morphs. (A)** TEM of three layers of chromatophores in a green individual. **(B)** Detailed structure of iridophores in a green individual. **(C)** TEM of three layers of chromatophores in a yellow individual. **(D)** Detailed structure of iridophores in a yellow individual.

**

**

**Fig. S2. Synteny tracker of chromosomes among genomes of *Naja naja*, *Thermophis baileyi,* *Ahaetulla prasina*, *Thamnophis elegans*, and** ***Zootoca vivipara*.** Synteny regions are colored according to chromosome of *Ahaetulla prasina* (Apra), to which they were collinear.

**
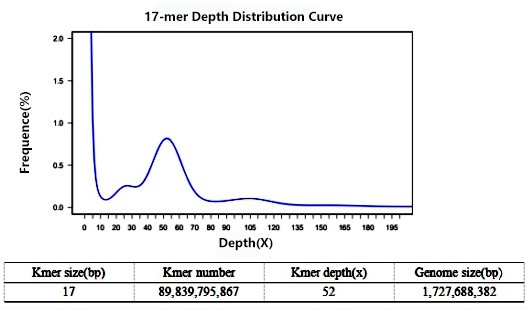
**

**Fig. S3. Genome complexity assessment.** The results showed that the genome heterozygosity and the ratio of duplicated sequences were low, and the *A. prasina* was diploid.

**
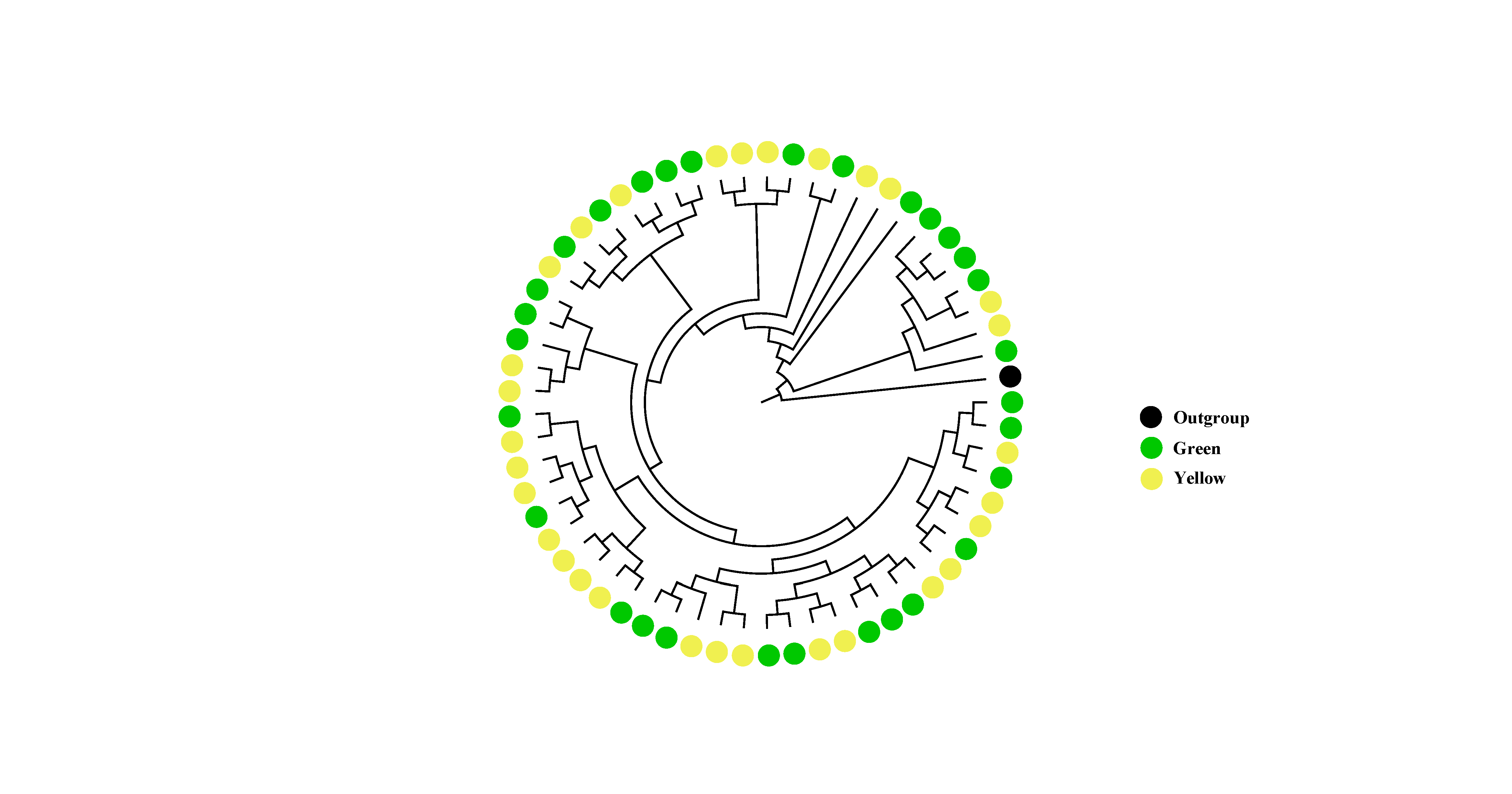
**

**Fig S4. Maximum-likelihood tree based on whole-genome SNPs.** The tree was rooted with an individual of *Thermophis baileyi* (outgroup).

**
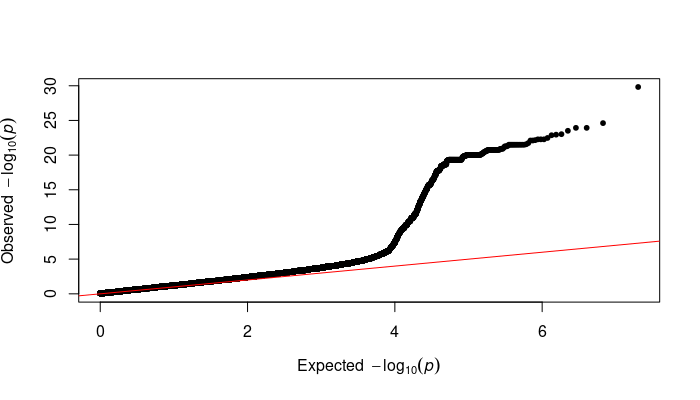
**

**Fig. S5. QQ-plot of all SNPs based on *p*-value in GWAS analysis.** The y-axis represents the logarithm of the observed *p*-value of the SNPs and the x-axis represents the logarithm of the expected uniformly distributed *p*-value.


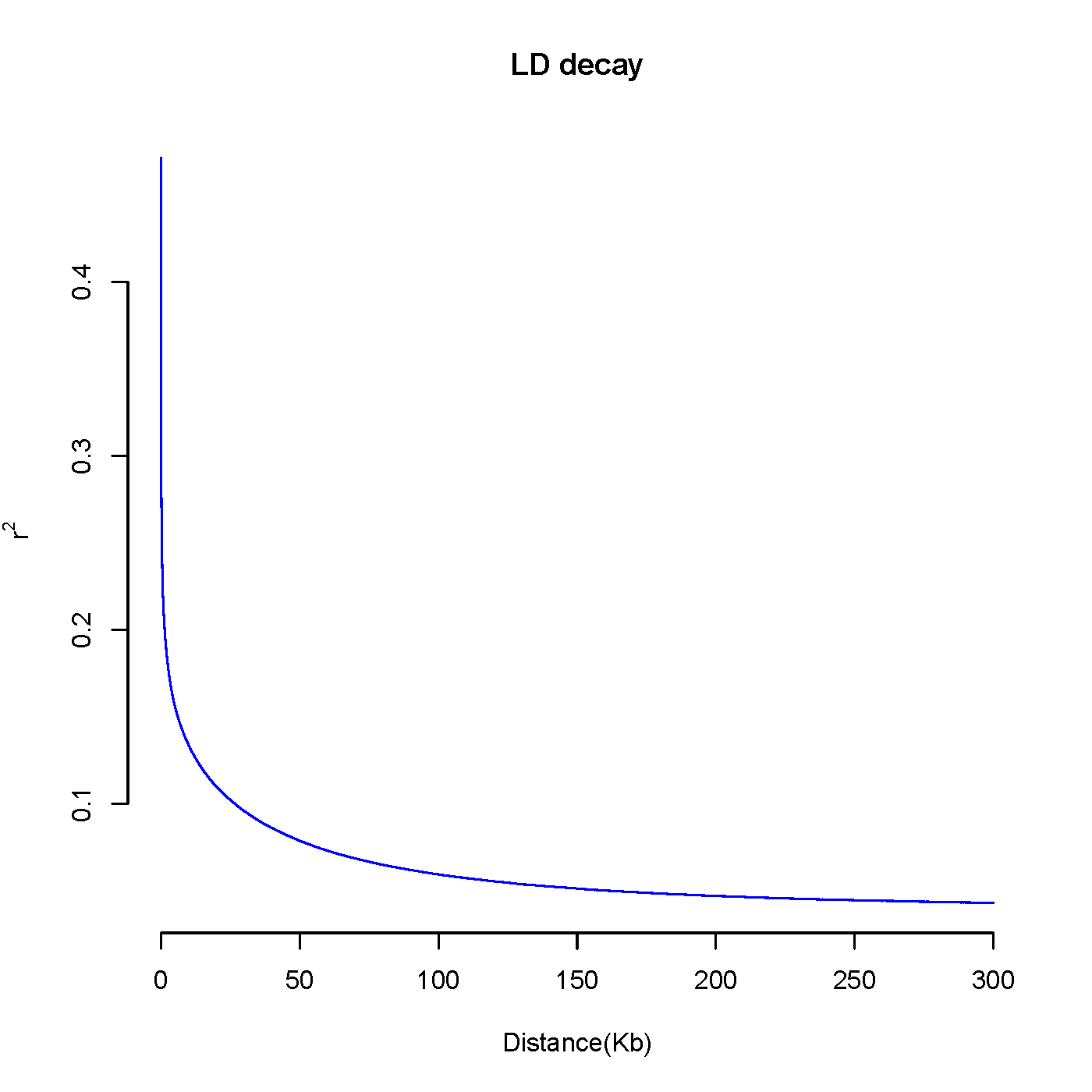


**Fig. S6. Linkage Disequilibrium decay rates of *A.prasina*.**

**
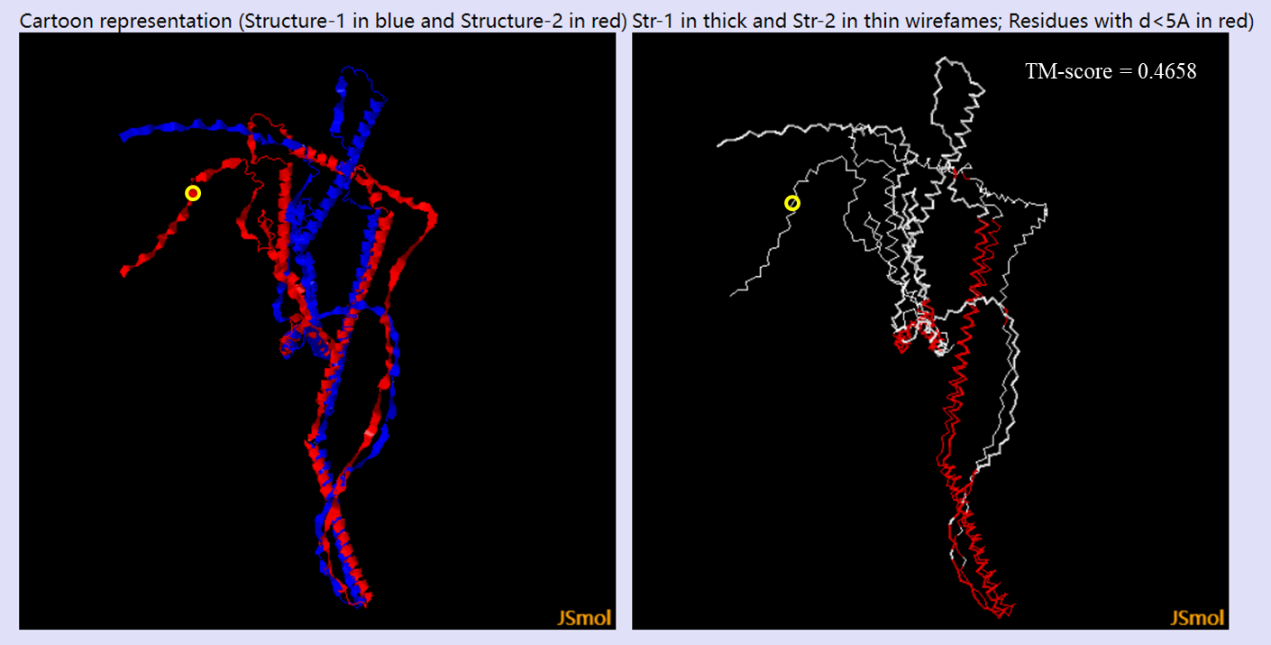
Fig. S7. The predicted spatial structure comparison between wild-type and mutant proteins of SMARCE1.** Left panel, wild-type was in blue and mutant in red; right panel, residues with d<5A were in red otherwise in white, the white residues were considered to be non-overlapping in space; the TM-score of 0.4658 (<0.5) revealed structural differences between the wild-type and mutant-modeled structures; the yellow circles represent the residue of missense mutation p.P20S.


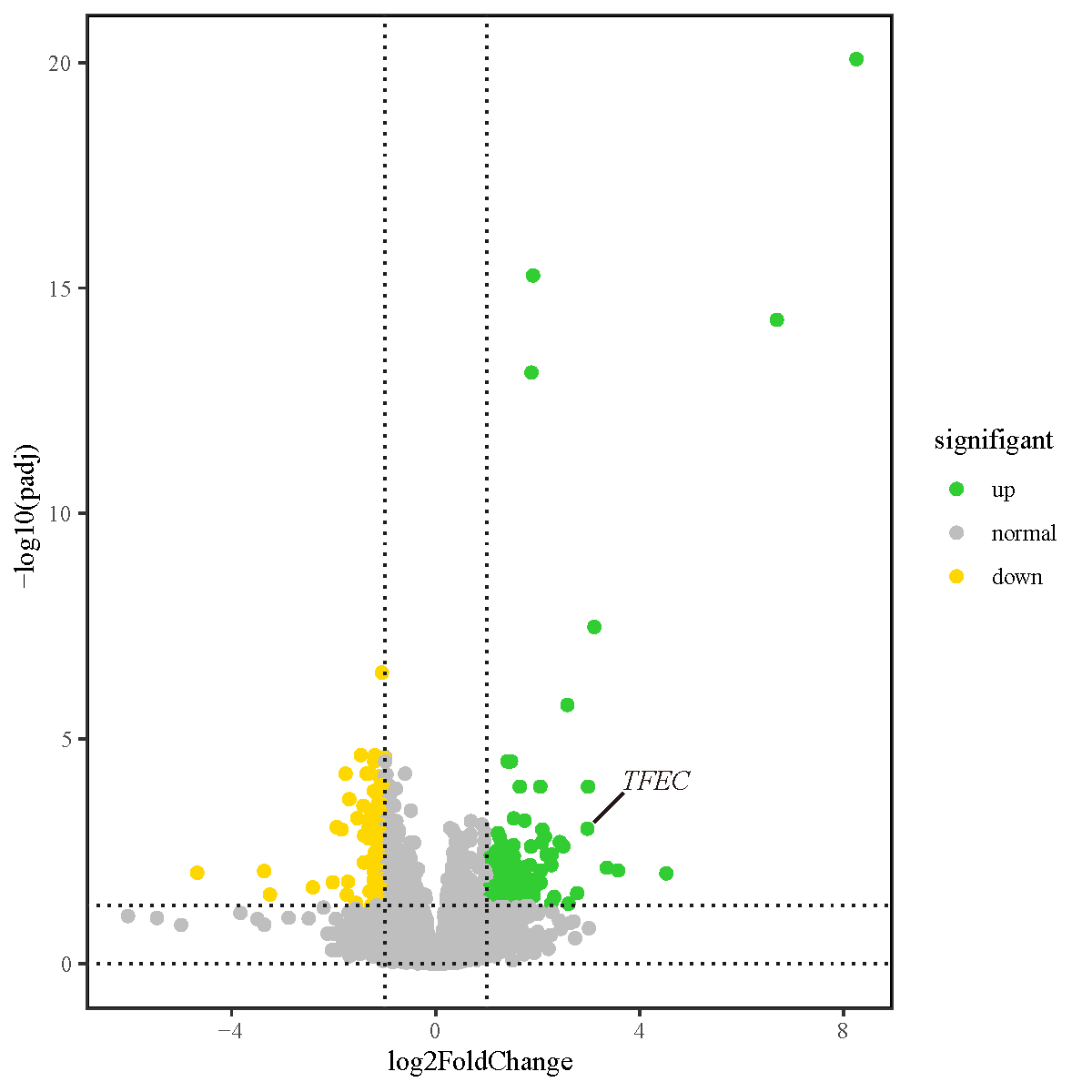


**Fig. S8. Volcano diagram of gene expression level.** Expression of 103 genes are up-regulated in green morphs and 102 are down-regulated. Expression level of *TFEC* was significantly higher in green morphs.


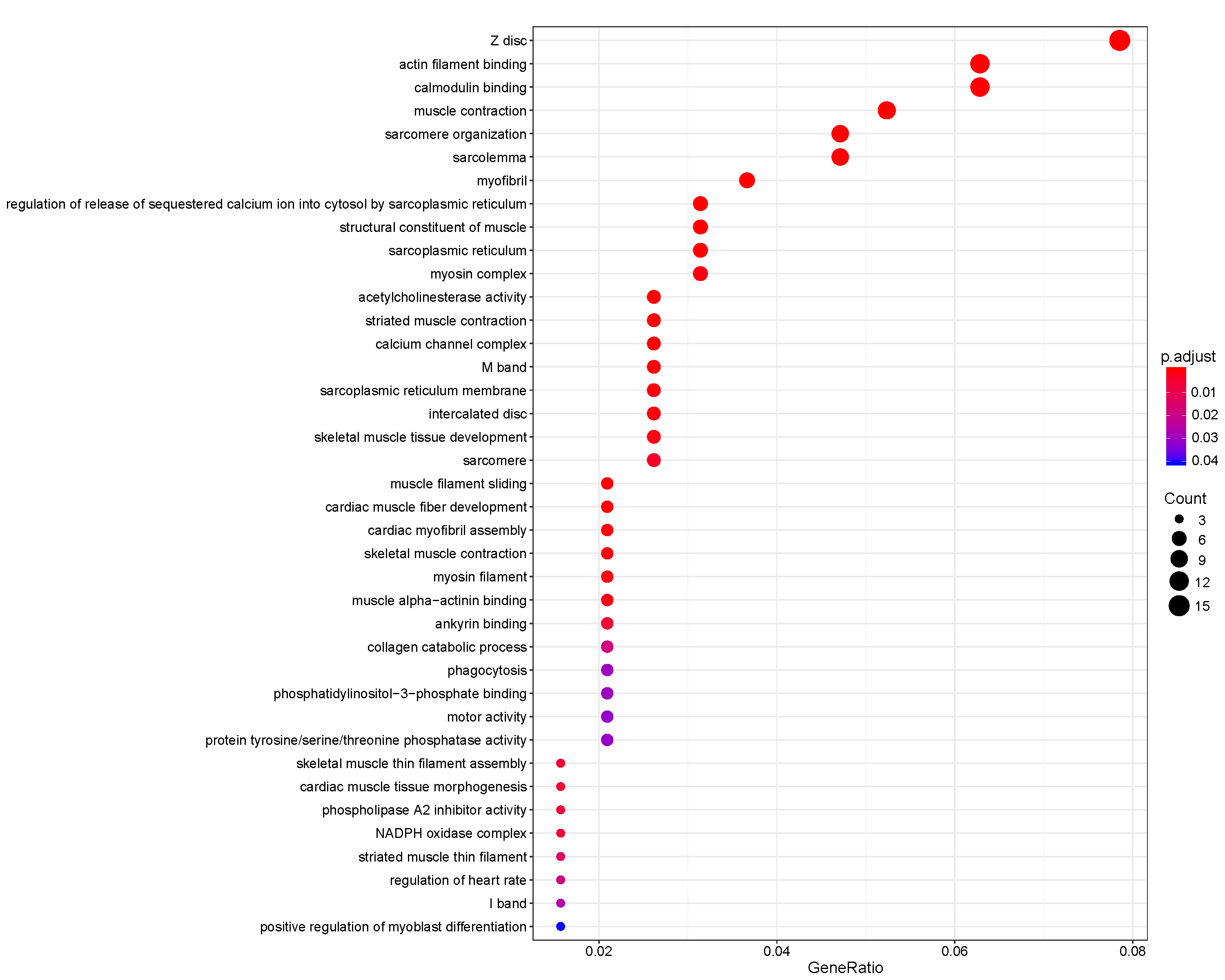


**Fig. S9. Dotplot of statistically significant GO terms enriched by DEGs.**


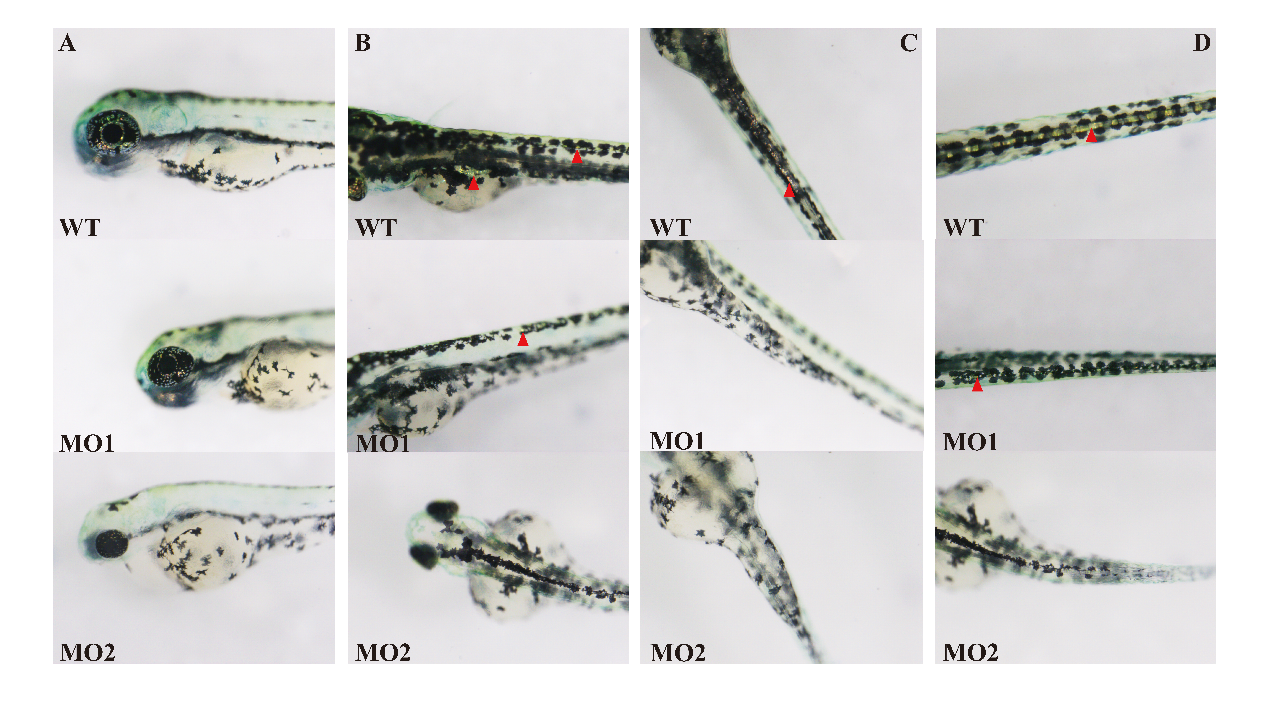


**Fig. S10. Morpholino injected and wildtype embryos at 72 hpf. (A)** Reduction of iridophores in MO embryos’ eyes, **(B)** dorsal, **(C)** ventral and **(D)** tail. Body curvature, cardiac edema and smaller eyes were observed obviously in MO2 group.

**
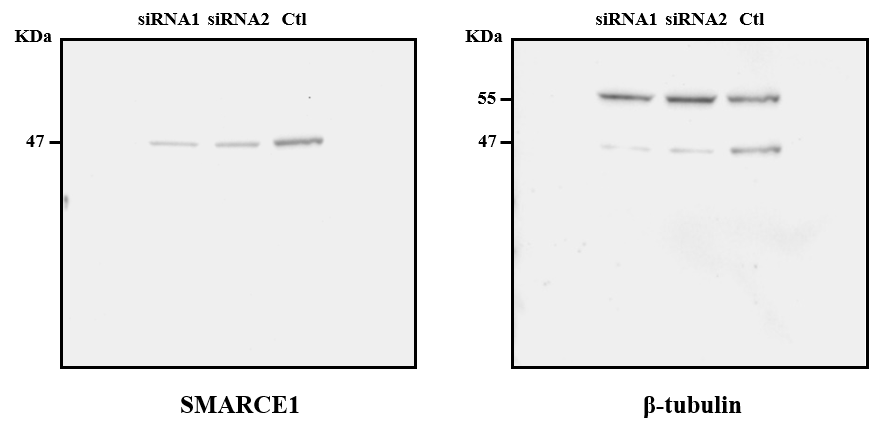
**

**Fig. S11. The uncropped western blot membranes that showed in Fig. 5B.** The mark in the second row of the right panel is the result of unwashed SMARCE1 on the same membrane.

**Supplementary Tables**

**Table S1. Information on the content of main chromatophore-related metabolites.**

| **Name** | **CIB110544*** | **CIB117715** | **CIB117707** | **CIB117581** | **CIB117646** | **CIB117583** | **FoldChange** | **Pvalue** |
| --- | --- | --- | --- | --- | --- | --- | --- | --- |
| Isoxanthopterin | 2.04E+08 | 1.98E+08 | 4.26E+08 | 1.53E+08 | 2.61E+08 | 3.41E+08 | 1.096215 | 0.832281 |
| Riboflavin | 2.8E+08 | 60756806 | 3.53E+08 | 1.72E+08 | 4.26E+08 | 1.61E+08 | 0.914464 | 0.745354 |
| Guanine | 4.25E+09 | 3.51E+09 | 3.89E+09 | 2.48E+09 | 1.99E+09 | 2.16E+09 | 1.758634 | 0.002796 |
| Hypoxanthine | 4.46E+09 | 1.71E+10 | 1.36E+10 | 6.19E+09 | 7.32E+09 | 9.07E+09 | 1.556675 | 0.539351 |

*CIB110544, CIB117715, and CIB117707 were yellow morphs; CIB117581, CIB117646, and CIB117583 were green morphs.

**Table S2. The chromosome length of *Ahaetulla prasina.***

| **CHR** | **Size** | **Scaf Num** |
| --- | --- | --- |
| LG01 | 377,630,096 | 41 |
| LG02 | 304,594,478 | 73 |
| LG03 | 231,952,326 | 31 |
| LG04 | 151,055,455 | 43 |
| LG05 | 139,992,370 | 26 |
| LG06 | 115,329,606 | 16 |
| LG07 | 103,430,753 | 8 |
| LG08 | 94,584,096 | 15 |
| LG09 | 35,554,624 | 15 |
| LG10 | 34,269,576 | 9 |
| LG11 | 32,047,439 | 38 |
| LG12 | 30,603,456 | 6 |
| LG13 | 26,942,173 | 1 |
| LG14 | 24,840,683 | 11 |
| LG15 | 19,292,983 | 2 |
| LG16 | 15,333,348 | 4 |
| LG17 | 14,242,732 | 9 |
| LG18 | 11,523,669 | 1 |
| Total | 1,763,219,863 | 349 |

**Table S3. Statistics for *Ahaetulla prasina* genome assemblies*.***

| **Stat Type** | **Contig Length（bp）** | **Contig Number** |
| --- | --- | --- |
| N50 | 23,873,608 | 23 |
| N60 | 16,934,886 | 32 |
| N70 | 12,055,984 | 44 |
| N80 | 9,723,955 | 60 |
| N90 | 5,801,550 | 84 |
| Longest | 85,847,614 | 1 |
| Total | 1,772,386,980 | 1,079 |
| Length>=1kb | 1,772,386,980 | 1,079 |
| Length>=2kb | 1,772,386,980 | 1,079 |
| Length>=5kb | 1,772,378,922 | 1,077 |

**Table S4. Statistics of the completeness genomes using BUSCO.**

| **Type** | **Number** | **Percent(%)** |
| --- | --- | --- |
| Complete BUSCOs | 2,400 | 92.8 |
| Complete and single-copy BUSCOs (S) | 2,382 | 92.1 |
| Complete and duplicated BUSCOs (D) | 18 | 0.7 |
| Fragmented BUSCOs (F) | 21 | 3.2 |
| Missing BUSCOs (M) | 104 | 4 |
| Total BUSCO groups searched | 2,586 | - |

**Table S5. Quality metrics for *A. prasina* genome compared to other published snake genomes.**

| **Species** | **Size (Gb)** | **Scaffold N50 (bp)** | **Contig N50 (bp)** | **BUSCO** |
| --- | --- | --- | --- | --- |
| ***Ahaetulla prasina*** | **1.77** | **231,952,326** | **23,873,608** | **92.80%** |
| *Boa constrictor* | 1.45 | 16,597,778 | 47,284 | 94.10% |
| *Python bivittatus* | 1.44 | 213,970 | 10,658 | 88.50% |
| *Notechis scutatus* | 1.67 | 5,997,050 | 31,763 | 86.60% |
| *Ophiophagus hannah* | 1.59 | 241,519 | 5,201 | 82.50% |
| *Pseudonaja textilis* | 1.59 | 14,685,528 | 50,443 | 87.40% |
| *Crotalus viridis* | 1.34 | 139,167 | 15,735 | 83.30% |
| *Naja naja* | 1.77 | 224,088,900 | 302,474 | 89.50% |
| *Hydrophis curtus* | 1.62 | 1,346,643 | 183,470 | 91.10% |
| *Deinagkistrodon acutus* | 1.47 | 2,122,253 | 22,424 | 91.10% |

**Table S6. Statistics of the annotated genes.**

| **Species** | **Number of genes** | **Average gene length (bp)** | **Average CDS length (bp)** | **Average exons per gene** | **Average exon length (bp)** | **Average introns per gene** | **Average intron length (bp)** |
| --- | --- | --- | --- | --- | --- | --- | --- |
| ***A.prasina*** | **18,362** | **39,682.96** | **1,729.16** | **10.31** | **210.87** | **9.31** | **4,027.21** |
| *O.hanna* | 18,445 | 20,606.77 | 1,255.51 | 7.63 | 164.62 | 6.63 | 2,920.11 |
| *P.bivittatus* | 19,793 | 24,683.61 | 1,580.05 | 9.21 | 171.55 | 8.21 | 2,813.86 |
| *T.sirtalis* | 18,565 | 23,028.66 | 1,366.34 | 7.6 | 179.85 | 6.6 | 3,283.59 |
| *P.mucros* | 20,001 | 25,428.94 | 1,558.63 | 9.18 | 169.83 | 8.18 | 2,919.03 |
| *P.textilis* | 19,358 | 31,020.84 | 1,655.50 | 9.65 | 171.58 | 8.65 | 3,395.38 |
| *N.scutatus* | 19,770 | 29,762.71 | 1,580.90 | 9.14 | 172.88 | 8.14 | 3,460.31 |

**Table S7. Overview of whole genome sequencing data.**

| **Voucher ID** | **Sample ID** | **Color** | **Locality** | **ReadSum** | **BaseSum** | **Q30(%)** |
| --- | --- | --- | --- | --- | --- | --- |
| CIB110500 | BN2018158 | green | Xishuangbanna, Yunnan, China | 284.0Mb | 42.6Gb | 93.18 |
| CIB110501 | BN2018159 | green | Xishuangbanna, Yunnan, China | 211.0Mb | 31.6Gb | 92.69 |
| CIB110502 | BN2018160 | yellow | Xishuangbanna, Yunnan, China | 211.2Mb | 31.6Gb | 91.06 |
| CIB110503 | BN2018161 | yellow | Xishuangbanna, Yunnan, China | 265.9Mb | 39.8Gb | 92 |
| CIB110504 | BN2018162 | yellow | Xishuangbanna, Yunnan, China | 213.4Mb | 32.0Gb | 92.18 |
| CIB110505 | BN2018163 | yellow | Xishuangbanna, Yunnan, China | 207.9Mb | 31.1Gb | 92.51 |
| CIB110506 | BN2018164 | yellow | Xishuangbanna, Yunnan, China | 208.8Mb | 31.3Gb | 92.57 |
| CIB110507 | BN2018165 | yellow | Xishuangbanna, Yunnan, China | 252.6Mb | 37.8Gb | 92.8 |
| CIB110508 | BN2018166 | yellow | Xishuangbanna, Yunnan, China | 212.8Mb | 31.9Gb | 92.86 |
| CIB110509 | YN2018426 | yellow | Xishuangbanna, Yunnan, China | 216.3Mb | 32.4Gb | 92.56 |
| CIB110510 | YN2018427 | yellow | Xishuangbanna, Yunnan, China | 226.7Mb | 34.0Gb | 92.75 |
| CIB110511 | YN2018428 | yellow | Xishuangbanna, Yunnan, China | 218.0Mb | 32.7Gb | 92.02 |
| CIB110512 | YN2018429 | yellow | Xishuangbanna, Yunnan, China | 212.0Mb | 31.8Gb | 92.67 |
| CIB110513 | YN2018430 | yellow | Xishuangbanna, Yunnan, China | 261.6Mb | 39.2Gb | 92.88 |
| CIB110514 | YN2018431 | green | Xishuangbanna, Yunnan, China | 209.3Mb | 31.4Gb | 93.02 |
| CIB110515 | YN2018432 | green | Xishuangbanna, Yunnan, China | 237.6Mb | 35.6Gb | 93.03 |
| CIB110516 | YN2018433 | green | Xishuangbanna, Yunnan, China | 322.2Mb | 48.3Gb | 92.5 |
| CIB110517 | YN2018434 | green | Xishuangbanna, Yunnan, China | 219.9Mb | 32.9Gb | 93 |
| CIB110518 | YN2018435 | green | Xishuangbanna, Yunnan, China | 330.6Mb | 49.5Gb | 93.07 |
| CIB110519 | YN2018436 | green | Xishuangbanna, Yunnan, China | 205.2Mb | 30.7Gb | 91.58 |
| CIB110520 | YN2018437 | green | Xishuangbanna, Yunnan, China | 322.0Mb | 48.3Gb | 93.04 |
| CIB110521 | YN2018438 | green | Xishuangbanna, Yunnan, China | 293.2Mb | 43.9Gb | 93 |
| CIB110529 | GJF2018001 | green | Xishuangbanna, Yunnan, China | 270.8Mb | 40.6Gb | 92.93 |
| CIB110530 | GJF2018002 | yellow | Xishuangbanna, Yunnan, China | 257.1Mb | 38.5Gb | 93.25 |
| CIB110533 | GJF2018003 | yellow | Xishuangbanna, Yunnan, China | 217.4Mb | 32.6Gb | 93.16 |
| CIB110534 | GJF2018004 | green | Xishuangbanna, Yunnan, China | 297.2Mb | 44.5Gb | 92.81 |
| CIB110536 | GJF2018005 | yellow | Xishuangbanna, Yunnan, China | 260.6Mb | 39.0Gb | 92.62 |
| CIB110537 | GJF2018006 | yellow | Xishuangbanna, Yunnan, China | 209.7Mb | 31.4Gb | 92.44 |
| CIB110538 | GJF2018007 | yellow | Xishuangbanna, Yunnan, China | 274.5Mb | 41.1Gb | 92.6 |
| CIB110539 | GJF2018008 | yellow | Xishuangbanna, Yunnan, China | 265.6Mb | 39.8Gb | 93.25 |
| CIB110540 | GJF2018009 | green | Xishuangbanna, Yunnan, China | 226.0Mb | 33.9Gb | 93.36 |
| CIB110541 | GJF2018010 | green | Xishuangbanna, Yunnan, China | 233.3Mb | 34.9Gb | 91.64 |
| CIB110543 | WW2019001 | yellow | Xishuangbanna, Yunnan, China | 103.7Mb | 31,1Gb | 93.12 |
| CIB110544 | WW2019002 | yellow | Xishuangbanna, Yunnan, China | 124.8Mb | 37.4Gb | 93.48 |
| CIB110545 | WW2019003 | yellow | Xishuangbanna, Yunnan, China | 163.9Mb | 49.2Gb | 93.23 |
| CIB110546 | WW2019004 | yellow | Xishuangbanna, Yunnan, China | 153.7Mb | 46.1Gb | 93.76 |
| CIB110547 | WW2019005 | yellow | Xishuangbanna, Yunnan, China | 148.4Mb | 44.5Gb | 92.95 |
| CIB110548 | WW2019006 | yellow | Xishuangbanna, Yunnan, China | 135.1Mb | 40.5Gb | 93.75 |
| CIB110549 | WW2019007 | yellow | Xishuangbanna, Yunnan, China | 100.8Mb | 30.2Gb | 92.76 |
| CIB110550 | WW2019008 | yellow | Xishuangbanna, Yunnan, China | 118.3Mb | 35.5Gb | 93.04 |
| CIB110551 | LAB-BN2019011 | yellow | Xishuangbanna, Yunnan, China | 111.8Mb | 33.5Gb | 93.61 |
| CIB110552 | LAB-BN2019012 | yellow | Xishuangbanna, Yunnan, China | 141.9Mb | 42.6Gb | 93.7 |
| CIB110553 | LAB-BN2019013 | green | Xishuangbanna, Yunnan, China | 146.8Mb | 44.0Gb | 93.9 |
| CIB110554 | LAB-BN2019014 | green | Xishuangbanna, Yunnan, China | 128.9Mb | 38.7Gb | 93.81 |
| CIB110555 | LAB-BN2019015 | green | Xishuangbanna, Yunnan, China | 130.2Mb | 39.1Gb | 93.14 |
| CIB110556 | LAB-BN2019016 | green | Xishuangbanna, Yunnan, China | 114.2Mb | 34.3Gb | 93.61 |
| CIB110557 | LAB-BN2019017 | green | Xishuangbanna, Yunnan, China | 144.7Mb | 43.4Gb | 93.48 |
| CIB110558 | LAB-BN2019018 | green | Xishuangbanna, Yunnan, China | 149.9Mb | 45.0Gb | 91.61 |
| CIB110559 | LAB-BN2019019 | green | Xishuangbanna, Yunnan, China | 135.0Mb | 40.5Gb | 93.62 |
| CIB110560 | LAB-BN2019020 | green | Xishuangbanna, Yunnan, China | 103.2Mb | 31.0Gb | 94.43 |
| CIB110561 | LAB-BN2019021 | green | Xishuangbanna, Yunnan, China | 107.1Mb | 32.1Gb | 92.99 |
| CIB110562 | LAB-BN2019022 | green | Xishuangbanna, Yunnan, China | 122.3Mb | 36.7Gb | 94.6 |
| CIB117581 | LAB2019023 | green | Xishuangbanna, Yunnan, China | 218.2Mb | 65.5Gb | 93.34 |
| CIB117646 | LAB2019024 | green | Yingjiang, Yunnan, China | 247.4Mb | 74.2Gb | 92.86 |
| CIB117583 | LAB2019025 | green | Yingjiang, Yunnan, China | 108.8Mb | 32.7Gb | 94.75 |
| CIB116269 | LAB2019268 | green | Yingjiang, Yunnan, China | 104.5Mb | 31.3Gb | 94.71 |
| CIB116308 | LAB2019269 | green | Yingjiang, Yunnan, China | 133.9Mb | 40.2Gb | 93.25 |
| CIB116305 | LAB2019272 | green | Yingjiang, Yunnan, China | 107.3Mb | 32.2Gb | 94.73 |
| CIB117715 | LAB2019311 | yellow | Yingjiang, Yunnan, China | 112.9Mb | 33.9Gb | 94.32 |
| CIB117707 | LAB2019312 | yellow | Yingjiang, Yunnan, China | 129.4Mb | 38.8Gb | 94.08 |

**Table S8. The top 30 genomic windows of population differentiation (Fst).**

| **CHR** | **Window position(start)** | **Window position(end)** | **Number of SNPs** | **Weighted Fst** |
| --- | --- | --- | --- | --- |
| 4 | 42,965,001 | 42,975,000 | 29 | 0.393592 |
| 4 | 43,005,001 | 43,015,000 | 58 | 0.374325 |
| 4 | 43,010,001 | 43,020,000 | 76 | 0.431505 |
| 4 | 43,015,001 | 43,025,000 | 103 | 0.379753 |
| 4 | 43,070,001 | 43,080,000 | 131 | 0.449459 |
| 4 | 43,075,001 | 43,085,000 | 91 | 0.455468 |
| 4 | 43,120,001 | 43,130,000 | 34 | 0.374188 |
| 4 | 43,125,001 | 43,135,000 | 31 | 0.388819 |
| 4 | 43,130,001 | 43,140,000 | 26 | 0.36951 |
| 4 | 43,190,001 | 43,200,000 | 40 | 0.400529 |
| 4 | 43,195,001 | 43,205,000 | 49 | 0.419589 |
| 4 | 43,200,001 | 43,210,000 | 52 | 0.437964 |
| 4 | 43,205,001 | 43,215,000 | 102 | 0.569729 |
| 4 | 43,210,001 | 43,220,000 | 157 | 0.668874 |
| 4 | 43,215,001 | 43,225,000 | 128 | 0.705509 |
| 4 | 43,220,001 | 43,230,000 | 126 | 0.704368 |
| 4 | 43,225,001 | 43,235,000 | 159 | 0.700259 |
| 4 | 43,230,001 | 43,240,000 | 144 | 0.697871 |
| 4 | 43,235,001 | 43,245,000 | 129 | 0.698762 |
| 4 | 43,240,001 | 43,250,000 | 97 | 0.674441 |
| 4 | 43,245,001 | 43,255,000 | 64 | 0.646624 |
| 4 | 43,250,001 | 43,260,000 | 46 | 0.646934 |
| 4 | 43,255,001 | 43,265,000 | 32 | 0.632402 |
| 4 | 43,260,001 | 43,270,000 | 20 | 0.612325 |
| 4 | 43,265,001 | 43,275,000 | 32 | 0.570324 |
| 4 | 43,270,001 | 43,280,000 | 44 | 0.52144 |
| 4 | 43,275,001 | 43,285,000 | 42 | 0.440138 |
| 4 | 43,280,001 | 43,290,000 | 62 | 0.412582 |
| 4 | 43,300,001 | 43,310,000 | 83 | 0.384667 |
| 4 | 43,335,001 | 43,345,000 | 22 | 0.377764 |

**Table S10. Coding genes within the region of GWAS signals.**

| **CHR** | **Position_Begin** | **Position_End** | **Gene** |
| --- | --- | --- | --- |
| Chr04 | 42,910,468 | 42,917,596 | *KRT15* |
| Chr04 | 42,925,290 | 42,927,254 | *uncharacterized* |
| Chr04 | 42,981,287 | 43,005,927 | *KRT23* |
| Chr04 | 43,041,241 | 43,054,903 | *KRT42* |
| Chr04 | 43,082,617 | 43,090,914 | *KRT25* |
| Chr04 | 43,093,704 | 43,112,247 | *KRT12* |
| Chr04 | 43,117,225 | 43,130,465 | *KRT12* |
| Chr04 | 43,140,828 | 43,150,095 | *KRT27* |
| Chr04 | 43,174,006 | 43,196,778 | *KRT222* |
| Chr04 | 43,217,268 | 43,254,171 | *SMARCE1* |
| Chr04 | 43,290,781 | 43,303,797 | *CCR7* |

**Table S11. Annotation of 3 missense variants using snpEFF software.**

| **CHR** | **Position** | **REF** | **ALT** | **Annotation** | **Putative_impact** | **Gene** | **HGVS.c*** | **HGVS.p*** |
| --- | --- | --- | --- | --- | --- | --- | --- | --- |
| Chr04 | 43,094,106 | G | A | missense_variant | MODERATE | *KRT12* | c.403G>A | p.Gly135Ser |
| Chr04 | 43,233,281 | C | T | missense_variant | MODERATE | *SMARCE1* | c.58C>T | p.Pro20Ser |
| Chr04 | 43,253,965 | C | T | missense_variant | MODERATE | *SMARCE1* | c.1033C>T | p.Pro345Ser |

*HGVS.c: Variant using HGVS notation (DNA level)

HGVS.p: If variant is coding, this field describes the variant using HGVS notation (Protein level).

**Table S12. Summary of protein variation effect prediction.**

| **Gene** | **substitution** | **PROVEAN score** | | **Prediction (Cutoff = -2.5)** | |
| --- | --- | --- | --- | --- | --- |
| *KRT12*  *SMARCE1*  *SMARCE1* | G135S  P20S  P345S | | 3.569  -6.331  -0.063 | | Neutral  Deleterious  Neutral |

**Table S13. Summary of RNA-seq data from 30 skin samples.**

| **Voucher ID** | **Sample ID** | **Color** | **Raw Reads** | **Raw Base(Gb)** | **Q20** | **Q30** |
| --- | --- | --- | --- | --- | --- | --- |
| CIB110500 | BN2018158 | green | 27,690,378 | 8.31 | 97.63 | 93.53 |
| CIB110501 | BN2018159 | green | 28,649,242 | 8.59 | 97.31 | 92.84 |
| CIB110502 | BN2018160 | yellow | 28,162,426 | 8.45 | 97.73 | 94 |
| CIB110503 | BN2018161 | yellow | 33,911,008 | 10.17 | 97.77 | 93.92 |
| CIB110505 | BN2018163 | yellow | 26,195,523 | 7.86 | 97.59 | 93.48 |
| CIB110511 | YN2018428 | yellow | 29,788,218 | 8.94 | 97.72 | 93.71 |
| CIB110513 | YN2018430 | yellow | 28,580,066 | 8.57 | 97.84 | 93.96 |
| CIB110515 | YN2018432 | green | 28,984,284 | 8.7 | 97.62 | 93.59 |
| CIB110518 | YN2018435 | green | 27,704,962 | 8.31 | 97.56 | 93.4 |
| CIB110520 | YN2018437 | green | 29,652,221 | 8.9 | 97.74 | 93.84 |
| CIB110530 | GJF2018002 | yellow | 29,202,466 | 8.76 | 97.68 | 93.63 |
| CIB110533 | GJF2018003 | yellow | 28,180,730 | 8.45 | 97.83 | 93.96 |
| CIB110540 | GJF2018009 | green | 31,890,662 | 9.57 | 97.42 | 93.13 |
| CIB110543 | WW2019001 | yellow | 26,273,452 | 7.88 | 97.69 | 93.66 |
| CIB110545 | WW2019003 | yellow | 27,845,510 | 8.35 | 97.78 | 93.81 |
| CIB110546 | WW2019004 | yellow | 26,927,949 | 8.08 | 97.75 | 93.82 |
| CIB110547 | WW2019005 | yellow | 27,613,615 | 8.28 | 97.42 | 93.06 |
| CIB110548 | WW2019006 | yellow | 29,958,020 | 8.99 | 97.46 | 93.2 |
| CIB110549 | WW2019007 | yellow | 26,177,415 | 7.85 | 97.72 | 93.76 |
| CIB110551 | LAB-BN2019011 | yellow | 27,590,240 | 8.28 | 97.66 | 93.62 |
| CIB110552 | LAB-BN2019012 | yellow | 27,195,043 | 8.16 | 97.53 | 93.27 |
| CIB110553 | LAB-BN2019013 | green | 28,885,779 | 8.67 | 97.48 | 93.2 |
| CIB110554 | LAB-BN2019014 | green | 28,018,746 | 8.41 | 97.8 | 93.94 |
| CIB110555 | LAB-BN2019015 | green | 29,058,269 | 8.72 | 97.75 | 93.84 |
| CIB110556 | LAB-BN2019016 | green | 28,980,855 | 8.69 | 97.75 | 93.88 |
| CIB110557 | LAB-BN2019017 | green | 29,350,749 | 8.81 | 97.75 | 93.74 |
| CIB110558 | LAB-BN2019018 | green | 28,413,202 | 8.52 | 97.66 | 93.56 |
| CIB110559 | LAB-BN2019019 | green | 28,854,936 | 8.66 | 97.66 | 93.53 |
| CIB110561 | LAB-BN2019021 | green | 27,119,130 | 8.14 | 97.77 | 93.78 |
| CIB110562 | LAB-BN2019022 | green | 27,825,948 | 8.35 | 97.79 | 93.83 |

**Table S14. Primers used in qRT-PCR.**

| **Name** | **Sequence(5'-3')** |
| --- | --- |
| SMARCE1_F | AGAGGAACGACACCAGGAGA |
| SMARCE1_R | CCTCTGCCTGTGCAATCTCA |
| Foxd3_F | CCACAACCTCTCACTCAACG |
| Foxd3_R | GGCTGTAAGCGCCGAA |
| β-actin_F | CTGGAACGGTGAAGGTGACA |
| β-actin_R | AAGGGACTTCCTGTAACAATGCA |
