# Supplementary material for: Genetic mapping and molecular mechanism behind color variation in the Asian vine snake
Source: Genome Biol. 2023 Mar 9;24:46. doi: 10.1186/s13059-023-02887-z (PMC9999515; doi:10.1186/s13059-023-02887-z)
Supplement: Supplementary file 3 — Additional file 3. Review history [file 13059_2023_2887_MOESM3_ESM.docx]

Review History

**First round of review**

**Reviewer 1**

**Were you able to assess all statistics in the manuscript, including the appropriateness of statistical tests used?** Yes, and I have assessed the statistics in my report.

**Comments to author:**

In this manuscript, the authors assemble and annotate a new chromosome-level genome for the Asian vine snake (Ahetulla prasina), and leverage this to explore genes that underlie color polymorphism in reptiles. To identify genes linked to color polymorphism, the conduct GWAS studies, mRNAseq studies, and follow-up functional studies (in fish) to identify evidence that an identified gene SMARCE1 (and an apparently functionally-related gene, tfec) may be important in chromatophore (iridophore) development and this relevant in coloration polymorphism. The authors do a reasonably convincing job of demonstrating evidence that mutations in SMARCE1 are very likely to result in the color variation observed in these snakes, and also linking this SMARCE1 variant to probable variation in the activity of another protein TFEC. Notably, unlike the well studied melanin-based melanophore coloration, reptilian coloration systems are far less well studied and less well understood; this study therefore fills important gaps in our broader understanding of genes linked to coloration vertebrates, including reptiles specifically, which have far more mechanisms and cell types that generate coloration and pattern.

Overall, I believe that the paper makes a number of strong and valuable contributions that argue for merit and publication in Genome Biology, however major revisions are required before it would be acceptable. For example, the contributions of the new chromosome-level genome, together with the very nice work on identifying the molecular basis of color variation makes this study a valuable contribution. And generally, I think the science and findings of the study are strong and merit publication, but in its current form, there are a very large number of English grammar issues that need to be corrected, that I would suggest require professional services outside of normal peer review.

Major Comments/Suggestions

Overall the manuscript is reasonably well-written (in terms of scientific content and overall narrative). However, the manuscript MUST undergo further careful review and revision for English grammar and proper English usage (e.g., proper tense, and context, comma usage, etc.) by a native English speaker (or service) to fix widespread issues with the text beyond what I have pointed out below (see examples in the Minor Comments section below). I would estimate that approximately one of every three sentence requires such revision. Also, the Introduction and Methods sections required the most substantial revisions for appropriate grammar, and contained the highest frequencies of errors, although the Discussion was in far better condition.

Minor comments, edits and suggestions:

* Figure 1A: while it looks sortof interesting, the many layers on this circus plot make it absolutely useless for interpretation. I would suggest just showing one or two tracks (e.g., like repeat content, or something simple) for the interior circles so at least there is some interpretation possible.

* LINE 285: I suggest starting a new paragraph with the sentence "According to GWAS,…" but modifying this first sentence to "Based on our GWAS analyses of color morphs, …"

* LINE 314: Revise this sentence, as it has no clear meaning as written currently.

* LINE 357: The authors should provide information on the localities of collection to the greatest extent possible, at least for the reference genome animal.

Examples of copy edits that need to be made throughout the entire manusceipt, but are too numerous to identify in standard peer review:

* LINE 79: Change "colorations" to "coloration"

* LINE 80: Change "Works have shown" to "Previous studies have demonstrated … "

* LINE 85: Change "chromatophores" to "chromatophore"

* LINE 90: this is an example of a sentence that needs more substantial review by professional writers/editors: "Neither determined by sex, season nor individual fitness [25], skin colors in Asian vine snake is speculated due to genetic variation". In this case, I don't think there is a clear scientific point being made that is important, and the best solution would be to delete this sentence.

* LINE 151: This sentence "We applied genome-wide association study (GWAS) and found an interval on chromosome 4 that was strongly…" needs to be rewritten to describe in an appropriate level of detail what was done and citing specific methods and precise results (e.g., which models, p-values, etc.).

* Line 175: please explain what is meant by this sentence more precisely. Assuming it refers to the two individuals that were heterozygous but with a yellow phenotype? Maybe also refer to Figure 3F?

* Line 194: does this sentence intend to say that TFEC is a "master REGULATORY gene"

**Reviewer 2**

**Were you able to assess all statistics in the manuscript, including the appropriateness of statistical tests used?** No, I do not feel adequately qualified to assess the statistics.

**Comments to author:**

The authors do a thorough job of testing the genetic, structural, and chemical differences that cause the difference in the two most common color morphs in Asian Vine Snakes, yellow and green. Overall, I think the conclusions are well supported and the figures highlight the results nicely.

My primary concern with the paper is the level of detail in the methods. Many of the tests, analyses, and techniques are not explained in detail. Additionally, sample sizes are not listed in the methods and it is not clear if the yellow and green snakes collected are from the same interbreeding population or if they are samples collected from many different places in southern China. I have highlighted some areas in the methods but the level of detail should be improved throughout. This can be sent to the supplemental document if there is not enough space to include it in the article itself.

I have included additional edits, comments, and suggestions throughout the manuscript in the attached PDF document. The ones I will reiterate here are:

1) Make sure to reference figures and tables in the discussion so that the reader knows which data support the claim when putting it in the context of the literature.

2) Tone down any statements that infer broader conclusions about color variation in the snake or other reptiles. Since only two color morphs were tested, there is still a lot of variation that is not accounted for in this study.

3) Figure S1 - write out the genus names for the species compared.

4) Table S5 - the last snake is written as "T. snake" which is not a scientific name. Also, the second column should have an 's' at the end of gene.

5) The GO term analysis and the connection to the results from the study are not clear to me. Please explain further in the discussion why the GO terms for muscle related genes is important in iridophores (Lines 311 - 315)

6) Need to discuss the other color morphs and what you expect to see in those based on this study and the other information known about reptile color.

7) Permit numbers and IACUC protocol numbers need to be included in the ethics statement at the beginning of the methods.

**Authors Response**

**Point-by-point responses to the reviewers’ comments:**

Reviewer 1:

**In this manuscript, the authors assemble and annotate a new chromosome-level genome for the Asian vine snake (Ahetulla prasina), and leverage this to explore genes that underlie color polymorphism in reptiles. To identify genes linked to color polymorphism, the conduct GWAS studies, mRNAseq studies, and follow-up functional studies (in fish) to identify evidence that an identified gene SMARCE1 (and an apparently functionally-related gene, tfec) may be important in chromatophore (iridophore) development and this relevant in coloration polymorphism. The authors do a reasonably convincing job of demonstrating evidence that mutations in SMARCE1 are very likely to result in the color variation observed in these snakes, and also linking this SMARCE1 variant to probable variation in the activity of another protein TFEC. Notably, unlike the well studied melanin-based melanophore coloration, reptilian coloration systems are far less well studied and less well understood; this study therefore fills important gaps in our broader understanding of genes linked to coloration vertebrates, including reptiles specifically, which have far more mechanisms and cell types that generate coloration and pattern.**

**Overall, I believe that the paper makes a number of strong and valuable contributions that argue for merit and publication in Genome Biology, however major revisions are required before it would be acceptable. For example, the contributions of the new chromosome-level genome, together with the very nice work on identifying the molecular basis of color variation makes this study a valuable contribution. And generally, I think the science and findings of the study are strong and merit publication, but in its current form, there are a very large number of English grammar issues that need to be corrected, that I would suggest require professional services outside of normal peer review.**

Thank you for your recognition of this work and many helpful comments. We have revised the manuscript according to your suggestions (please see our point-by-point responses below). Further, the manuscript has been polished by a native English speaker. We hope our detailed responses below and revision meet with your approval.

**Major Comments/Suggestions**

**Overall the manuscript is reasonably well-written (in terms of scientific content and overall narrative). However, the manuscript MUST undergo further careful review and revision for English grammar and proper English usage (e.g., proper tense, and context, comma usage, etc.) by a native English speaker (or service) to fix widespread issues with the text beyond what I have pointed out below (see examples in the Minor Comments section below). I would estimate that approximately one of every three sentence requires such revision. Also, the Introduction and Methods sections required the most substantial revisions for appropriate grammar, and contained the highest frequencies of errors, although the Discussion was in far better condition.**

Thank you for your detailed advice. We have improved the language throughout the manuscript and have had the manuscript polished by a professional native English-speaking editor.

**Minor comments, edits and suggestions:**

1. **Figure 1A: while it looks sortof interesting, the many layers on this circus plot make it absolutely useless for interpretation. I would suggest just showing one or two tracks (e.g., like repeat content, or something simple) for the interior circles so at least there is some interpretation possible.**

Thank you for your constructive suggestions. We retained the circular rings referring to coding genes of the plus (blue) and minus (yellow) strands, and GC content per 100-kb windows, respectively. We have revised the figure below and added interpretation details in the revised manuscript.

2. **LINE 285: I suggest starting a new paragraph with** **the sentence "According to GWAS,…" but modifying this first sentence to "Based on our GWAS analyses of color morphs, …"**

Thank you for your suggestions. We have revised the sentence in line 290 as follows: “Based on our GWAS analyses of color morphs, we found a conservative missense mutation…”

3. **LINE 314: Revise this sentence, as it has no clear meaning as written currently.**

Thank you for your advice. We have revised the sentence in line 316 as follows: “Notably, functional enrichment analysis also identified changes in the chromatophores of the Asian vine snakes. As actin and myosin filaments play roles in multiple chromatophore cell processes, such as motility, morphological changes, and structural organization of iridophores [54, 55], the DEGs-enriched functions between the two morphs provide further clues regarding different iridophore morphogenesis (Fig. S9)”.

4. **LINE 357: The authors should provide information on the localities of collection to the greatest extent possible, at least for the reference genome animal.**

Thank you for your valuable comments. We have added the localities of collections in Table S7. And the reference genome snake was from Xishuangbanna, Yunnan, China (added in line 409).

5. **LINE 79: Change "colorations" to "coloration"**

Corrected.

6. **LINE 80: Change "Works have shown" to "Previous studies have demonstrated … "**

Corrected.

7. **LINE 85: Change "chromatophores" to "chromatophore"**

Corrected.

8. **LINE 90: this is an example of a sentence that needs more substantial review by professional writers/editors: "Neither determined by sex, season nor individual fitness [25], skin colors in Asian vine snake is speculated due to genetic variation". In this case, I don't think there is a clear scientific point being made that is important, and the best solution would be to delete this sentence.**

Thank you for your comments. We have deleted this sentence in the revised manuscript.

9. **LINE 151: This sentence "We applied genome-wide association study (GWAS) and found an interval on chromosome 4 that was strongly…" needs to be rewritten to describe in an appropriate level of detail what was done and citing specific methods and precise results (e.g., which models, p-values, etc.).**

Thank you for your valuable suggestions. We have revised the sentence in line 150 as follow: “We applied a genome-wide association study (GWAS) using Fisher’s exact test with a Bonferroni-corrected *p* < 0.05 threshold. We identified an interval on chromosome 4 that contained 903 genome-wide significant SNPs (-log10 *p* = 8.40) and showed a strong association with the color phenotype (Fig. 3A), with high confidence (Fig. S5)”

10. **Line 175: please explain what is meant by this sentence more precisely. Assuming it refers to the two individuals that were heterozygous but with a yellow phenotype? Maybe also refer to Figure 3F?**

We apologize for the confusion. Indeed, the sentence describes the two yellow individuals with heterozygous loci (C/T) (as shown in Fig. 3F). We have revised the sentence in line 174 as follow: “The two heterozygous yellow-skinned snakes exhibited locus discordance, possibly due to incomplete penetrance or other interacting genetic factors.”

11. **Line 194: does this sentence intend to say that TFEC is a "master REGULATORY gene"**

Thank you for your comments. Indeed, *TFEC* is a reported master regulatory gene of iridophore differentiation and chromatophore development. Our transcriptome results further defined its different expression in two color morphs of Asian vine snakes, suggesting differences in chromatophore development. We have revised the sentence in line 194 as follow: “The significant difference in *tfec* expression further highlighted the crucial role of chromatophore development in color variation between the yellow and green morphs.”

Reviewer 2:

**The authors do a thorough job of testing the genetic, structural, and chemical differences that cause the difference in the two most common color morphs in Asian Vine Snakes, yellow and green. Overall, I think the conclusions are well supported and the figures highlight the results nicely.**

**My primary concern with the paper is the level of detail in the methods. Many of the tests, analyses, and techniques are not explained in detail. Additionally, sample sizes are not listed in the methods and it is not clear if the yellow and green snakes collected are from the same interbreeding population or if they are samples collected from many different places in southern China. I have highlighted some areas in the methods but the level of detail should be improved throughout. This can be sent to the supplemental document if there is not enough space to include it in the article itself.**

Thank you for your recognition of our work and detailed comments. We have revised the manuscript according to your suggestions (please see our point-by-point responses below). We hope our detailed responses below and revision meet with your approval.

1. **Make sure to reference figures and tables in the discussion so that the reader knows which data support the claim when putting it in the context of the literature.**

Thank you for your suggestion. We have added figure and table references in the Discussion section.

2. **Tone down any statements that infer broader conclusions about color variation in the snake or other reptiles. Since only two color morphs were tested, there is still a lot of variation that is not accounted for in this study.**

Thank you for your comments. We have modified the corresponding parts of the manuscript, e.g., line 184: “These findings strongly suggest that *SMARCE1* p.P20S is a crucial mutation in the color variation between green and yellow morphs of Asian vine snakes.” and line 248: “…we investigated the molecular mechanisms of color variation in Asian vine snakes to suggest a possible genetic basis of vivid reptile coloration.”

3. **Figure S1 - write out the genus names for the species compared.**

Thank you for your advice. We have added the names in the description.

4. **Table S5 - the last snake is written as "T. snake" which is not a scientific name. Also, the second column should have an 's' at the end of gene.**

We apologize for the error. We have modified the species name and changed ‘gene’ to ‘genes’.

5. **The GO term analysis and the connection to the results from the study are not clear to me. Please explain further in the discussion why the GO terms for muscle related genes is important in iridophores (Lines 311 - 315)**

We apologize for the confusion. As discussed in this part, muscle constructions such as actin and myosin play vital roles in chromatophore development, especially the morphogenesis of iridophores (Li et al., 2015; Rohrich et al., 1974). The differential expression of related genes in our analyses between the two different color morphs may indicate changes in the morphogenetic process of iridophores. We have clarified the discussion in line 316 as follows: “Notably, functional enrichment analysis also identified changes in the chromatophores of the Asian vine snakes. As actin and myosin filaments play roles in multiple chromatophore cell processes, such as motility, morphological changes, and structural organization of iridophores [54, 55], the DEGs-enriched functions between the two morphs provide further clues regarding different iridophore morphogenesis (Fig. S9).”

6. **Need to discuss the other color morphs and what you expect to see in those based on this study and the other information known about reptile color.**

Thank you for your valuable advice. Actually, according to our hypothesis, blue Asian vine snakes may have similar but denser and smaller guanine crystal morphology as green-skinned snakes, with fewer xanthophores (reflecting shorter wavelength light). Whereas brown and gray (off-white) skinned snakes may have looser and more disordered crystals as found in yellow skins (reflecting longer wavelength light). As in previous research, there were thicker and more disordered crystal platelets, longer distances between platelets in iridophores of white/red/brown lizard skins than in blue/green skins (Kuriyama et al., 2017; Kuriyana et al., 2016; Saenko et al., 2013). The morphology of reflective crystals in iridophores may underlie the formation of different body colors in Asian vine snakes. In Squamate reptiles, the formation of skin color is a complex process involving interactions of multiple chromatophores (Saenko et al., 2013). The top layer of xanthophores and bottom layer of melanophores can absorb shorter wavelengths of blue-violet light and longer wavelengths of red-orange light, respectively (Bagnara et al., 2007). Subtle differences in xanthophore and melanophore morphology may be a potential reason for the formation of the other three skin colors rather than green and yellow. The diversity of body color in reptiles is due to variant morphology of reflecting crystals in iridophores, as well as differences in the content and arrangement of xanthophores and melanophores. In addition, based on our hypothesis, the same *SMARCE1* mutation detected in yellow morphs may be discovered in brown skins (with similar iridophore morphology as yellow morphs) but not in blue skins. Additional samples and experiments are needed to validate the mechanism of chromatophores in Asian vine snakes.

7. **Permit numbers and IACUC protocol numbers need to be included in the ethics statement at the beginning of the methods.**

Thank you for your helpful advice. We have added the numbers and revised the sentence in line 348-354 as follows: “The authors followed all appropriate ethical and legal guidelines and regulations. The Chengdu Institute of Biology, Chinese Academy of Sciences (CIB, CAS), facilitated the collection and exportation permits necessary for this and related studies. The study was conducted according to the guidelines of the Declaration of Helsinki and was approved by the Animal Ethics Committee of Chengdu Institute of Biology, Chinese Academy of Sciences (CIBDWLL20202632, 13 April 2020). Research procedures were carried out in accordance with all national and institutional regulations.”

We have revised the corresponding words and phrases according to all suggested edits in the PDF document from the reviewer. The following are compilations and responses to the remaining recommendations in the attached PDF document:

8. **Line 38: Paper and figures seem to indicate melanophores are also important.**

Thank you for your comment. Indeed, some differences in the melanophores were observed based on the TEM results. However, iridophores were the main basis of skin color differences in the Asian vine snakes (for changing hues). We discussed the possible melanin differences in the Discussion section (Line 269). In addition, we have revised the sentence in line 39 as follows: “…chromatophore morphology (mainly iridophores) was the main basis of skin color difference.”

9. **Line 47: With one color variant (since only yellow and green were tested).**

Corrected.

10. **Line 52: The meaning of the second half of this sentence is unclear. Should it be "to the environment as well as survival and reproduction for organisms"?**

We apologize for the confusion. We have revised the sentence in line 54 as follows: “Phenotypic diversity of organisms is the basis of environmental adaptation, as well as survival and reproduction.”

11. **Line 55: This is not made clear for the present study.**

Thank you for your comment. We have revised the sentence in line 55 as follows: “Color polymorphism is an important component of phenotypic diversity, defined as the presence of two or more morphs with different color phenotypes (color variants) within a population [1].”

12. **Line 67: This suggests chromatophores are skeletal components. Is that correct? If not, reword sentence.**

We apologize for the error. We have modified the sentence in line 66 as follows: “NCCs are multipotent embryonic cells that give rise to a vast array of derivatives, including neurons, skeletal components, and chromatophores [7].”

13. **Line95: What about the other 3 color morphs?**

As other color morphs are rare and it is difficult to obtain sufficient samples, we could not include them in the experimental design at the time. As seen in our response to comment #6, based on our research, we speculate that the complex mechanism underlying the other three skin colors involves the three chromatophores. If sufficient samples can be obtained in the future, we will consider continuing the study in-depth.

14. **Line 110: There is no indication of how many individuals this was done on. If more than one individual, did all the yellow individuals look the same?**

We apologize for the error. We have added sample information in line 360. All yellow skins tested showed the same chromatophore morphology. We have added the required images in the supplementary file (Fig. S1).

15. **Line 120: I would like to see their function discussed in the discussion more thouroughly.**

Thank you for your comments. We have replaced the relevant downstream products (IMP) with direct metabolite (hypoxanthine) content to illustrate the differences in iridophores more clearly. Guanines and hypoxanthine are the main components of light-reflected platelets in iridophores. Rich purine content predicts more and larger reflective platelet crystals, which can change the wavelength of reflected lights. Thus, the significantly higher guanine content (fold-change = 1.76) in our metabolite analysis indicated larger reflective crystals in the yellow morphs. Higher hypoxanthine content (fold-change = 1.56) may further indicate differences in crystals. We have added the metabolite information in Table S1 and revised the discussion in line 259 as follows: “Multiple thin-layer interference occurs in the reflective platelet layers mainly composed of guanine and hypoxanthine crystals in iridophores [17]. Higher purine content results in larger crystal reflective platelets in iridophores that reflect longer wavelength light [36, 37]. Guanine and hypoxanthine as major differential metabolites in the two different skin morphs, may indicate differences in iridophores in the Asian vine snakes (Fig. 1I, Table S1).” Description of the results has also been revised in line 118 as follows: “In agreement with the TEM results, we found no significant differences in the content of carotenoids and pterins, but guanine was significantly higher in the yellow morphs, with higher hypoxanthine content (Fig. 1I, Table S1).”

16. **Line 150: Were the snakes collected from two populations (i.e., locations) or are you refering to the color morphs as distinct populations?**

We apologize for the confusion. We have added the localities of collections in Table S7. All snakes were collected from Yunnan Province, China, and both color morphs co-exist at the same location. In our analyses at the time, we divided all samples into two groups based on color to compare the linkage disequilibrium (LD) decay, and used the results as the window size parameter in Fst calculation to reduce the impact of gene linkage effects. However, now, it seems more appropriate to make the calculation for all samples and we have re-calculated the LD decay rates in the revised manuscript (obtained the same results as previously). We have replaced Fig. S6 with a suitable figure and revised the descriptions in line 155 as follows: “Genomic scans for population genetic differentiation (measured by Fst) were conducted using a sliding window of 10 kb length based on the Linkage disequilibrium (LD) decay rates (Fig. S6).”

17. **Line 185: Can't state this because it has already been established that there are other color morph variants. Since they were not included in the analysis, the conclusions are limited to differentiating the green and yellow morphs.**

We apologize for the overstatement. We have modified the sentence in line 184 as follows: “These findings strongly suggest that SMARCE1 p.P20S is a crucial mutation in the color variation between green and yellow morphs of Asian vine snakes.”

18. **Line 196: It is not clear to me how the two sentences are connected. Hopefully it is discussed more in the discussion.**

As discussed in our response to Comment 5, actin and myosin filaments play critical roles in multiple cellular processes of chromatophores (e.g., formation of crystal array architecture in iridophores). The DEGs-enrichment results of actin- and myosin-related terms suggested changes in developmental processes of chromatophores, especially iridophores, between the two color morphs. We have improved the discussion in line 316 as follows: “Notably, functional enrichment analysis also identified changes in the chromatophores of the Asian vine snakes. As actin and myosin filaments play roles in multiple chromatophore cell processes, such as motility, morphological changes, and structural organization of iridophores [54, 55], the DEGs-enriched functions between the two morphs provide further clues regarding different iridophore morphogenesis (Fig. S9).”

19. **Line 207: Does this mean that only 20% were successfully/correctly injected? Does this also mean that 80% of the injected embryos had the same eye appearance as wild-type fish?**

We apologize for the confusion. This sentence means that all 156 embryos were correctly injected, 32 of which showed obviously distinct phenotypes compared to the wild-type embryos. We have revised the sentence in line 208 as follows: “However, 32 of the 156 injected embryos did not show obvious iridophore differentiation at the same stage (Fig. 4B, C).”

20. **Line 238: No figures/tables are referenced throughout the discussion to point to the data that supports the conclusions being made**

Thank you. We have added figure and table references in the Discussion section according to your advice.

21. **Line 247: This is a large overstatement. Finding one SNP in two morphs of one species doesn't resolve color polymorphism in all of reptiles.**

We apologize for the overstatement. We have revised the sentence in line 248 as follows: “…we investigated the molecular mechanisms of color variation in Asian vine snakes to suggest a possible genetic basis of vivid reptile coloration.”

22. **Line 255: Reference figure.**

Added.

23. **Line 264: Can you hypothesize about the other color morphs using this idea and the comparison to chameleons?**

Panther chameleons shift hue by actively tuning the guanine crystal lattice within a superficial thick layer of iridophores. When panther chameleons get excited, the arrangement of nanocrystals changes and causes iridophores to shift reflective light from short to long wavelengths, causing a corresponding shift from green to yellow/orange skin. According to our hypothesis, blue Asian vine snakes may have a similar guanine crystal stage as the resting state in panther chameleons (green/blue). Blue Asian vine snakes may have similar but denser and smaller guanine crystal morphology as green-skinned snakes, with fewer xanthophores (reflecting shorter wavelength blue-violet light). However, brown and gray (off-white) skins may have similar guanine crystal morphology as the excited state in panther chameleons (yellow/white), with looser and more disordered crystals, as found in the yellow snakes (reflecting longer wavelength light).

24. **Line 292: I think this sentence is referencing Figure S5. As a reader that is familiar with genomics and transcriptomics, I think this figure could be interpreted more on. If there was no difference in protein structure, would the red and blue strands be perfectly aligned everywhere?**

Thank you for your comments. The sentence is referring to Fig. S5 (Fig. S7 in the revised manuscript), and we have added the figure reference. TM-score was used as an indicator for assessing topological similarity in protein structure. It has a value in the [0,1] range, where 1 indicates all strands are perfectly aligned between two structures, and structures with an overall TM-score higher than 0.5 assume generally the same fold. Typically, the residue pairs of distance < 5.0A were considered to be spatially very close and they would be shown in red in the figure. In the right panel of Fig. S7, most paired residues were distant (colored in white, including p.P20S), suggesting structural differences between the wild-type and mutant proteins. We have revised the description of Fig. S7 as follows: “…the white residues are considered to be non-overlapping in space; the TM-score of 0.4658 (<0.5) revealed structural differences between the wild-type and mutant-modeled structures…”

25. **Line 321: Sometimes this is written in italics, sometimes not. That happens for the other genes.**

We apologize for the error. We have revised these issues throughout the manuscript.

26. **Line 327: How could your hypothesis explain the other color morphs in the species?**

As seen in our response to comment #6, based on our results and hypothesis, gray (off-white) and brown morphs could be considered as a similar group to yellow morphs as they may have similar reflecting-platelet crystal morphology, thus reflecting longer wavelength light. Both morphs possibly share the same p.P20S mutation in *SMARCE1*, leading to abnormal *TFEC* function in chromatophore differentiation, similar to the possible mechanism of the yellow morphs. In contrast, blue morphs may be similar to green morphs (wildtype), with their iridophore platelets reflecting shorter wavelength light. Vivid color polymorphism in reptiles arises due to complex interactions among the three chromatophores. For these three morphs, changes of xanthophore and melanophore morphology may likewise influence the final formation of skin color, which may be caused by other genes involved in chromatophore development or undetected mutations. Additional data and analyses are warranted to reveal the molecular mechanisms underlying other skin colors.

27. **Line 344: Include permit numbers and exportation numbers where appropriate and possible.**

Added in line 348-354.

28. **Line 345: Add the protocol number that was approved. Need one for both the snakes and the fish.**

Added and revised in line 353.

29. **Line 349: How large of skin samples?**

The size of samples has been added in line 360 as follow: “For the TEM experiments, two fresh skin samples (1cm×1cm) per color morph from the Asian vine snake were collected.”

30. **Line 349: How many total? how many per morph? where the specimen deposited in a natural history museum afterwards? Where they all from the same location? There needs to be a lot more informaiton about the animals used since you are comparing two groups (yellow/green). If they are not all from the same location, then geographic variation may be responsible.**

Thank you for your comments. We have added detailed information on sample collection in the corresponding sections of the manuscript and in Table S7. We have revised in line 358: “All vouchers are stored in the Herpetological Museum of the Chengdu Institute of Biology, Chinese Academy of Sciences.”

All specimens were collected from Yunnan Province, China. We calculated Fst values to evaluate genetic distances of all samples. The analysis was repeated 10 times by randomly dividing all samples into two groups of equal numbers and calculating the Fst values using VCFtools. As show in the table below, all tests indicated minimal genetic differentiation (≈0) within the samples. A phylogenetic tree based on whole-genome SNPs was also constructed to study the phylogenetic relationships between the two color morphs. The tree (see below) revealed that color phenotype had no effect on genetic structure and both color morphs co-existed. We have added the phylogenetic tree results in line 147.

| Test No. | Fst |
| --- | --- |
| 1 | -0.00399 |
| 2 | -0.00336 |
| 3 | -0.00421 |
| 4 | 0.00048 |
| 5 | -0.00334 |
| 6 | 0.002394 |
| 7 | -0.00368 |
| 8 | -0.00382 |
| 9 | 0.000159 |
| 10 | -0.00346 |

31. **Line 350: There is not enough detail here for this to be repeatable. The information about any equipment that was used needs to be included as well as anything about the process. If it is a standard protocol, please include citations to papers that detail it. I would suggest that TEM potentially has its own section.**

Thank you. We have added detailed information on methods according to your advice and have included TEM analysis as a separate section in line 360-370.

32. **Line 352: This also needs more detail so that it can be repeated. The HPLC column should be stated, the A and B phase, etc. Then, the information for the machines used.**

We apologize for the confusion. We have added detailed information on HPLC-MS/MS analysis according to your advice in line 373-405.

33. **Line 374: To make the methods clearer, there should be a paragraph/section on long-read DNA sequencing and a paragraph/section on short-read DNA sequencing. There is no indication of sample size in the methods thus far.**

Thank you for your advice. We have clarified the ‘Genome sequencing’ section in line 408-438.

34. **Line 404: Before sequencing, you have to isolate mRNA and convert it to cDNA. All of those details are missing.**

Thank you for your comments. All missing information has been added in line 459-462 as follows: “RNA was extracted from the heart, lung, and kidney using TRIzol reagent (Invitrogen) for all samples. PolyA mRNA was isolated using oligonucleotide (dT) magnetic beads and disrupted into short segments, and cDNA was synthesized using random hexamer primers and reverse transcriptase.”

35. **Line 404: Why was skin not used?**

Thank you for your comment. The main reason is that RNA quality of snake skin samples for reference genome sequencing was not qualified for RNA sequencing at the time. Even so, according to our genome annotation and transcriptome analyses, we correctly obtained the transcripts and expression data of genes related to chromatophore development (e.g., *tfec*), which was evaluated not to affect the results of the current analysis.

36. **Line 423: What species? GeMoMa annotation is only as good as the input annotations.**

The related species were: *Ophiophagus hannah*, *Python bivittatus*, *Thamnophis sirtalis*, *Protobothrops mucrosquamatus*, *Pseudonaja textilis*, and *Notechis scutatus* (as shown in Table S6). In the present study, we combined the genomic data of the above six snakes for annotation using GeMoMa, which led to better annotation results. We have added the information in line 480-482 as follows: “…align the homologous peptides from related species (*Ophiophagus hannah, Python bivittatus, Thamnophis sirtalis, Protobothrops mucrosquamatus, Pseudonaja textilis, and Notechis scutatus,* Table S6) to the assembly…”

37. **Line 437: Will these be available in the link below?**

The sentence means that we used an in-house Perl script to retrieve GO annotations from the database below based on our SwissProt BLASTP results. We can upload the script to the public database for access if required.

38. **Line 439: This link does not work.**

We apologize for the error. The link to the database file presented was available at the time. However, the link is indeed currently inaccessible (probably due to maintenance). Another link to this database (https://ftp.uniprot.org/pub/databases/uniprot/knowledgebase/idmapping/) is currently accessible, but it is a different version to the file used in our analysis. If required, we can upload the file downloaded to the public database for access. To avoid ambiguity, we have revised the sentence to: “The SwissProt BLASTP results were processed using our in-house Perl script to retrieve associated GO terms from the idmapping database.”

39. **Line 449: Does that mean there were 60 total?**

Yes, this means 30 samples per morph (60 in total). We have revised the sentence in line 506 as follows: “Tail-tip tissue samples of 30 yellow and 30 green morphs were collected and high-quality DNA was extracted using a QIAGEN® Genomic kit.”

40. **Line 461: Illumina.**

Corrected.

41. **Line 511: Illumina.**

Corrected.

42. **Line 533: More detail needed.**

Thank you. We have added detailed information in line 587 as follows: “At different post-fertilization stages, the wild-type (WT) and morpholino (MO) embryo groups were imaged using a microscope (Nikon SMZ18, Japan), including chromatophores in the eyes, dorsal, and ventral.”

43. **Line 534: How?**

Thank you. We have revised the sentence in line 589 as follows: “We compared the chromatophore density of these three parts between two groups at the same stage and in the same field of view based on microscopy imaging.”

44. **Line 547: Incomplete sentence.**

Thank you. We have completed the sentence in line 602 as follows: “Caco2 cells were cultured at 37 °C under 5% CO_2_ in Dulbecco’s Modified Eagle Medium/Nutrient Mixture F-12 (DMEM/F12) (Gibco, USA) and 10% fetal bovine serum (FBS)”

45. **Fig. 3B: What about the** **other region on chromosome 4 that is enriched?**

The other six SNPs within other regions on Chr04 reached the threshold were removed because they were located in intergenic regions. Their effects need to be validated in follow-up studies.

46. **Fig.3G: Baileyi should not be capitalized. Also, it may be worth using common names for the species instead of scientific names so that more readers can recognize the species used in comparison.**

Thank you for your comments. According to your advice, we have replaced all scientific names with common names in Fig. 3G.

47. **Fig. 5B: B-actin control not shown.**

Thank you. The control group results have been added to Fig. 5B. We have also replaced the image with a clearer one.

48. **Fig. 5E: Deficient?**

Corrected.

**References**

Li XM, Song YN, Xiao GB, Zhu BH, Xu GC, Sun MY, Xiao J, Mahboob S, Al-Ghanim KA, Sun XW, Li JT. Gene Expression Variations of Red-White Skin Coloration in Common Carp (*Cyprinus carpio*). Int J Mol Sci. 2015; 16:21310-21329.

Rohrlich ST. Fine structural demonstration of ordered arrays of cytoplasmic filaments in vertebrate iridophores. A comparative survey. The Journal of cell biology. 1974; 62:295-304.

Kuriyama T, Esashi J, Hasegawa M. Light reflection from crystal platelets in iridophores determines green or brown skin coloration in *Takydromus* lizards. Zoology (Jena). 2017; 121:83-90.

Kuriyama T, Morimoto G, Miyaji K, Hasegawa M. Cellular basis of anti‐predator adaptation in a lizard with autotomizable blue tail against specific predators with different colour vision. J Zool. 2016; 300:89-98.

Saenko SV, Teyssier J, van der Marel D, Milinkovitch MC. Precise colocalization of interacting structural and pigmentary elements generates extensive color pattern variation in *Phelsuma* lizards. BMC Biol. 2013; 11:105.

Teyssier J, Saenko SV, van der Marel D, Milinkovitch MC. Photonic crystals cause active colour change in chameleons. Nat Commun. 2015; 6:6368.

Bagnara JT, Fernandez PJ, Fujii R. On the blue coloration of vertebrates. Pigment Cell Res. 2007; 20:14-26.

**Second round of review**

**Reviewer 2**

The authors have done an exceptional job responding to reviewer comments and feedback. My only two comments are to 1) include a collection permit number if they have one from the appropriate authorities in Yunnan Province and 2)if they so choose, add the information form their responses on the other color morphs - response 6, 23 and 26. These are in the response to reviewers but not added in the paper.

igure S8 - change "high-regulated" to "up-regulated"

Figure S9 - change "statistical" to "statistically"

Table S6 - "Mucros" is capitalized and it should be lower case. Also, N. scutatus has a space between the period and epithet and the others do not.

Response 37 - I will defer to the editor on the policy for deposition of scripts.

Reponse 38 - I don't think the file needs to be made accessible, rather, just the date of download would be sufficient.

**Authors Response**

**Point-by-point responses to the reviewers’ comments:**

The authors have done an exceptional job responding to reviewer comments and feedback. My only two comments are to 1) include a collection permit number if they have one from the appropriate authorities in Yunnan Province.

*Response: Thank you for your recognition of our work and detailed comments. The Asian vine snake is not a protected species in China, and all samples in our study were not collected from natural reserves, so that government approval was unnecessarily required for sample collection. All samples involved were obtained from the collection of the Herpetological Museum of the Chengdu Institute of Biology, Chinese Academy of Science over the years according to the requirements*.

2) if they so choose, add the information form their responses on the other color morphs - response 6, 23 and 26. These are in the response to reviewers but not added in the paper.

*Response: Thank you for your suggestion. We have summarized speculations on possible mechanisms for other color variants and added in the end of the discussion section: “In addition, we speculated that Asian vine snakes with other colors similarly follow this pattern of differences in iridophore morphology, and subtle differences in xanthophore and melanophore morphology may be the potential mechanisms for the formation of the other skin colors rather than green or yellow. Further researches are needed to verify the possibilities.”*

1.Figure S8 - change "high-regulated" to "up-regulated"

*Response: Corrected.*

2.Figure S9 - change "statistical" to "statistically"

*Response: Corrected.*

3.Table S6 - "Mucros" is capitalized and it should be lower case. Also, N. scutatus has a space between the period and epithet and the others do not.

*Response: Corrected.*

4.Response 37 - I will defer to the editor on the policy for deposition of scripts.

*Response: Thank you for your comments. We have uploaded the perl script to Github and Zenedo public database, and added information and links to it in the Availability of Data and Materials section of the manuscript as required: “…Perl scripts used for data analyses conducted as part of this study are available at Github: https://github.com/bioinformaticspcj/coding_gene_function_annotation/releases/tag/v1.0.0 [94] and Zenodo: https://doi.org/10.5281/zenodo.7549964 [95].”*

5.Reponse 38 - I don't think the file needs to be made accessible, rather, just the date

of download would be sufficient.

*Response: Thank you for your comments. We have added the date of download.*
